# TIRAP-mediated activation of p38 MAPK in inflammatory signaling

**DOI:** 10.1038/s41598-022-09528-8

**Published:** 2022-04-04

**Authors:** Sajjan Rajpoot, Ashutosh Kumar, Kam Y. J. Zhang, Siew Hua Gan, Mirza S. Baig

**Affiliations:** 1 Department of Biosciences and Biomedical Engineering (BSBE), Indian Institute of Technology Indore (IITI), Simrol, Indore, 453552 India; 2 Laboratory for Structural Bioinformatics, Center for Biosystems Dynamics Research, RIKEN, 1-7-22 Suehiro, Tsurumi, Yokohama, Kanagawa 230-0045 Japan; 3 School of Pharmacy, Monash University Malaysia, Jalan Lagoon Selatan, Selangor Darul Ehsan, Building 2, Level 5, Room 40 (2-5-40), 47500 Bandar Sunway, Malaysia

**Keywords:** Computational biology and bioinformatics, Pathogenesis

## Abstract

The role of TIRAP (toll/interleukin-1 receptor (TIR) domain-containing adapter protein) in macrophage inflammatory signalling has been significantly evolved since its discovery in 2001 due to its dynamic nature and subcellular localization to regulate multiple signaling through several protein–protein interactions (PPIs). Structural analysis of these interactions can reveal a better understanding of their conformational dynamics and the nature of their binding. Tyrosine phosphorylation in the TIR domain of TIRAP is very critical for its function. In toll-like receptor (TLR) 4/2 signalling, Bruton's tyrosine kinase (BTK) and Protein kinase C delta (PKCδ) are known to phosphorylate the Y86, Y106, Y159, and Y187 of TIRAP which is crucial for the downstream function of MAPKs (mitogen-activated protein kinases) activation. The objective of this study is to understand the interaction of TIRAP with p38 MAPK through molecular docking and identify the importance of TIRAP tyrosine phosphorylation in p38 MAPK interaction. In this structural study, we performed an in-silico molecular docking using HADDOCK 2.4, pyDockWEB, ClusPro 2.0, and ZDOCK 3.0.2 tools to unravel the interaction between TIRAP and p38 MAPK. Further, manual in-silico phosphorylations of TIRAP tyrosines; Y86, Y106, Y159, and Y187 was created in the Discovery Studio tool to study the conformational changes in protein docking and their binding affinities with p38 MAPK in comparison to non-phosphorylated state. Our molecular docking and 500 ns of molecular dynamic (MD) simulation study demonstrates that the Y86 phosphorylation (pY86) in TIRAP is crucial in promoting the higher binding affinity (∆G_bind_) with p38 MAPK. The conformational changes due to the tyrosine phosphorylation mainly at the Y86 site pull the TIRAP closer to the active site in the kinase domain of p38 MAPK and plays a significant role at the interface site which is reversed in its dephosphorylated state. The heatmap of interactions between the TIRAP and p38 MAPK after the MD simulation shows that the TIRAP pY86 structure makes the highest number of stable hydrogen bonds with p38 MAPK residues. Our findings may further be validated in an in-vitro system and would be crucial for targeting the TIRAP and p38 MAPK interaction for therapeutic purposes against the chronic inflammatory response and associated diseases.

## Introduction

Macrophages are crucial immune cells responsible for the initiation, activation, and resolution of inflammatory signalling in the host defensive system. They perform extensive roles in providing first-line defense through recognition of vast intra- and extracellular stimuli such as damaged cells or various extracellular pathogens patterns through their specialized pattern recognizing receptors (PRRs)^1–4^. The group of Toll-like receptors (TLRs) is one of the important members of PRRs which play critical roles in mediating host innate immune response and help in the initiation of the adaptive immune response. The activation of TLR4 in macrophages in response to its extracellular stimulus such as lipopolysaccharide (LPS) triggers its ectodomain dimerization and conformational changes in its cytoplasmic Toll/Interleukin-1 receptor (TIR) domain allowing it to initiate the recruitment of TIR domain-containing adaptor proteins. The process subsequently activates several downstream protein kinases, transcription factors such as AP-1 and NF- κB, and eventually the production of inflammatory cytokines^5–10^.

Out of the five known adaptor proteins, toll/interleukin-1 receptor domain-containing adapter protein **(**TIRAP) is identified as one of the most vital and actively involved TIR domain-containing proteins in TLR4/2 signalling pathways^11–14^. The initiation of intracellular signalling through TIRAP is complex and involves multiple protein–protein interactions through its TIR domain^15^. Structurally, protein phosphorylation is one the most vital and common post-translational modifications occurring in the majority of signal transduction in response to a robust stimulus. Similarly, tyrosine phosphorylation of TIRAP is crucial for its function and interaction with its partner protein^15–17^. In mammalian cells, the phosphor-proteomic analysis suggests that although most proteins undergo phosphorylation, some contain only a few phosphorylation sites, and some proteins have most of their serine, threonine, and tyrosine residues phosphorylated. Among the proteins, serine is by far, the most phosphorylated residue (86%), followed by phosphorylation of threonine (12%) and tyrosine residues (2%)^18,19^. However, tyrosine phosphorylation serves a plethora of functions and in phosphor-tyrosine signalling, the protein–protein interaction is one important module. Accumulating study suggest that the phospho-tyrosine binding module possess substrate or ligand recognition specificity to narrow down the potential interacting partners^20–23^.

The TIR domain of TIRAP contains a total of six tyrosine residues (Y86, Y106, Y159, Y187, Y194, and Y195) where the initial four significantly undergo phosphorylation modification, leading to the regulation of the downstream response^15,17^. In the presence of LPS, macrophages activate many tyrosine kinases including Src, Hck, and Lyn, acting on TLRs^16,24^. However, a study has revealed the presence of a tec family kinase burton’s tyrosine kinase (BTK) as a key tyrosine kinase that regulates the phosphorylation of TIRAP at tyrosine Y86, Y106, Y159, and Y187 including TLR4, TLR2, and MyD88^25–27^. In addition, protein kinase C delta (PKCδ) has also been established to act upon the tyrosine residues of TIRAP for its phosphorylation^28^. The effect of tyrosine phosphorylation on TIRAP is observed during the downstream signalling which regulates its interaction with MyD88, IRAK-2, TRAF6, leading to activation of MAPKs, mainly on p38 MAPK and transcription factor NF-kB^15,29–37^.

A dominant-negative response has been observed in LPS-stimulated macrophages signalling where tyrosine mutant TIRAP fails to induce the downstream kinase p38 MAPK and transcription factors such as NF-κB activation as compared to the wild-type^17^. In macrophages-mediated inflammatory response, p38 MAPK is a major player whose expression is upregulated besides undergoing tyrosine phosphorylation^36,37^. The p38 MAPK family consists of four subtypes (p38α MAPK, p38β MAPK, p38γ MAPK, and p38δ MAPK)^37,38^. As with TIRAP, the p38 MAPK is critical in LPS-induced macrophage signalling and its targeted deletion impairs cytokines expression^39^. From a disease perspective, the evidence suggests that p38 MAPK mediates the decisive role in inflammation of lung, kidney, liver, and brain and is termed as a critical player in macrophages mediated inflammatory diseases such as gastritis, colitis, arthritis, etc.^37,38^ Like TIRAP, the tyrosine residue of the p38 MAPK is also crucial for its activation and macrophages stimulation leads to the high expression of its active form of p38 MAPK phosphorylated at a tyrosine residue. The p38 MAPK subtypes are also expressed in other cells like endothelial cells, neutrophils as well as CD4^ +^ T cells. Structurally, the 38 kDa of p38 MAPK is comprised of a 135 amino acid long, secondary β-sheets N-terminal domain and a 225 amino acid long, secondary α-helical C-terminal domain while the catalytic site is located in between adjoining these two domains. The catalytic site contains a 13 amino acid phosphorylation lip from leucine (L171) to valine (V183) in which the phosphorylation of threonine (T180) and tyrosine (Y182) activates the p38 MAPK^37,40^. The activated p38 MAPK in stimulated macrophages leads to the production of several pro-inflammatory cytokines such as IL-1β, TNF-α, IL-12, COX-2, IL-8, IL-6, etc. which is implicated with the inflammatory diseases as mentioned above as well as others like granuloma, diabetes, and acute lung inflammation^37,41^. Therefore, several studies have signified the p38 MAPK as a crucial anti-inflammatory target, and a range of natural and chemical inhibitors are investigated such as AMG-548, SC-80036, SC-79659, quercetin, mycoepoxydiene, losmapimod, etc. and few also went through clinical trials^37,38,42,43^.

Besides their role in inflammation, the p38 MAPK has also an imperative role in cell proliferation, differentiation, and apoptosis^37,38^. The p38 MAPK is activated by different environmental stimuli and cellular stress and hence understanding their molecular mechanism of activation is more beneficial for deciding the target in disease-specific conditions, developing novel drugs, and countering the diseases effectively. In this *in-silico* study, the structural changes and impact of TIRAP tyrosines phosphorylation on p38 MAPK interaction are analyzed. The effect of phosphorylation of each tyrosine residue of TIRAP mentioned above and the complex conformational changes leading to a better binding affinity for interaction with p38 MAPK was also investigated to understand the interaction that may be important to understand its activation and when designing anti-inflammatory drugs.

## Materials and methods

### 3D structure preparation and molecular docking of TIRAP and p38 MAPK

Molecular docking of TIRAP and p38 MAPK crystal structures was performed to investigate their protein–protein interaction. The solved crystal structures of the TIRAP TIR domain (PDB ID 3UB2) and p38 MAPK (PDB ID 1WBV) were obtained from the RCSB PDB database (https://www.rcsb.org/) and were prepared for the docking studies. Both structures were prepared by removing the water, heteroatoms, and any co-crystallized ligand groups followed by the addition of polar hydrogen atoms. The prepared structure with a single chain for the TIRAP TIR domain in 3UB2 or p38 MAPK in 1WBV was finally saved in a PDB file format. The protein–protein molecular docking of TIRAP and p38 MAPK was performed in four docking platforms which includes HADDOCK 2.4^44^, pyDockWEB^45^, ClusPro 2.0^46^, and ZDOCK 3.0.2^47^ for confirmation. The interaction interface was analyzed in chimera v1.13.1 tool^48^. Finally, the images of the interactions were prepared in a Discovery Studio Visualizer v21.1.0.20298^49^.

### In-vitro cell culture for immunofluorescence staining and confocal microscopy

RAW 264.7 murine macrophage cell line was obtained from the cell repository of the National Centre for Cell Science (NCCS), Pune, India. For cellular colocalization of TIRAP and p38 MAPK in macrophages, RAW 264.7 murine macrophages cells (1 × 10^4^) were seeded on sterile coverslips in the well plate and grown for 24 h in Dulbecco’s minimal essential medium (DMEM) (11,965,118, Gibco) complete media supplemented with heat-inactivated 10% fetal bovine serum (FBS) (10,270,106, Gibco) and 1% penicillin–streptomycin (15,140,122, Gibco) in a humidified incubator with 5% CO_2_ at 37 °C. Before the experiment, the cells were washed with sterile 1X phosphate-buffered saline (PBS) at room temperature (RT) and replenished with fresh DMEM complete media. For immunofluorescence study, cells were stimulated with 250 ng/ml of lipopolysaccharide (LPS; Sigma L2630) for 1 h. After treatment, cells were rinsed twice with PBS and fixed in 4% paraformaldehyde for 15 min at RT. Cells were again washed thrice with PBS (3 × 5 min), and then permeabilized in 0.1% Triton X-100 in PBS for 10 min at RT. After three more washing, cells were then blocked with 5% bovine serum albumin (BSA) prepared in PBST (0.1% Tween-20 in PBS) for 1 h at RT. After blocking, the cells were briefly washed with PBST and incubated in the primary antibodies at 4 °C overnight. The anti-TIRAP (sc-166149, Santa Cruz Biotechnology, Inc.) and anti-p38 MAPK (9212, CST) were used in 1:200 dilution in PBST. After three washing with PBST, cells were incubated in secondary antibody for 1 h in dark using the goat anti-mouse Alexa Fluor 488 (A11001, Invitrogen) and chicken anti-rabbit Alexa Fluor 594 (A21442, Invitrogen) at 1:200 dilution in PBST. Finally, cells were washed thrice and mounted on glass slides over fluoro-shield mounting media containing DAPI for nuclear staining (F6057, Sigma) and stored at 4 °C for imaging. The confocal microscopy was performed using Olympus confocal laser scanning microscope (FV 1200 MPE Olympus) at 20X 2.5z and 100X 2.0z magnification. For colocalization analysis, ImageJ^50^, and its plugin JACoP^51^ were used. The data were plotted using GraphPad Prism 7.0 (GraphPad Software, USA) and presented as mean ± SEM while Student’s -test was performed for comparing two groups.

### In-silico TIRAP phosphorylation/dephosphorylation and molecular docking

The site-specific phosphorylation of tyrosine residue was obtained in the crystal structure of the TIRAP TIR domain (PDB 3UB2) through the Discovery Studio tool. The tyrosine residue at positions Y86, Y106, Y159, and Y187 were replaced with the phosphorylated tyrosine residue pY86, pY106, pY159, and pY187, and the files were saved in PDB formats. Single tyrosine as well as all four-tyrosine phosphorylated (pYall04) TIRAP structures were prepared. For sequential dephosphorylation study, TIRAP structure was generated with one non-phosphorylated tyrosine at a time followed by the remaining three phosphorylated conformations and subsequently, renamed as dpY86/pYall03, dpY106/pYall03, dpY159/pYall03, and dpY187/pYall03. All these PDB files of structures were energetically minimized in a Chimera v1.13.1 tool. The molecular docking of phosphorylated tyrosine TIRAP and non-phosphorylated p38 MAPK (PDB 1WBV) was performed in a HADDOCK 2.4^44^ supporting the docking of phosphorylated residues.

### Molecular dynamic (MD) simulation analysis

The TIRAP and p38 MAPK protein structures as well as the tyrosine-phosphorylated and non-phosphorylated TIRAP molecular docking complexes with p38 MAPK were selected as the initial starting point for the MD simulation study. The TIRAP and p38 MAPK protein structures as well as each complex were placed into a dodecahedron box before being solvated in TIP3P water molecules. The MD simulation system was neutralized by adding Na + or Cl − ions. CHARMM 27 force field was employed to assign parameters for the protein, solvent, and ion molecules^52^. The parameters for phosphorylated tyrosine were obtained from SwissSideChain database (https://www.swisssidechain.ch). Classical MD simulation was performed using GROMACS program v5.0.4 following typical energy minimization, equilibration, and production runs^53^. Initially, 50,000 steps of steepest-descent energy minimization were performed to relax the MD system. Energy minimization was followed by 1 ns equilibration under NVT and NPT ensemble respectively. Finally, 500 ns production MD runs were performed at a constant temperature of 300 K for each system. The simulations were performed using a time step of 2 fs. A cut-off of 10 Å was used for short-range interactions while the long-range interactions were handled using Particle-Mesh Ewald (PME) method. MDAnalysis^54^, MDTraj^55^, and scikit-learn^56^ python libraries were used to process and analyze MD trajectories. Principal component analysis (PCA) was performed using MODE-TASK program^57^*.* Intermolecular contacts were analyzed from the MD simulation trajectories using GetContacts (https://getcontacts.github.io/). Graphics were prepared using Matplotlib python library^58^, R^59^, and PyMOL v2.3.4^60^*.*

## Results and discussion

### TIR domain of TIRAP interacts with p38 MAPK

In the recent past, the TIRAP crucial role in TLR4 and TLR2 inflammatory signalling pathway has been investigated beyond its adaptor protein function. It is experimentally established that TIRAP interacts with several other proteins other than MyD88, which results in regulation of the downstream signalling as well as the activation of NF-kB and AP-1 transcription factor^15,30^. Based on our prior research work^34^, the structural conformation and mechanism of TIRAP to interact and regulate the downstream p38 MAPK, which is mainly responsible for the activation of transcription factors in inflammatory pathways, has been investigated here.

TIRAP protein, mostly known as an adaptor protein for TLR4 and TLR2, has two domains regulating its overall activity. Besides the N-terminal PBD (PIP2 binding domain) domain governing its subcellular location, the TIR domain is most crucial for TIRAP activity to regulate the interactions and/or signalling. Since many previous reports of TIRAP interaction with other proteins demonstrate the involvement of the TIR domain in all known interactions, we sought to understand its binding pattern with p38 MAPK. The *in-silico* approach was applied to establish the protein complex of TIRAP and p38 MAPK as well as to determine the best binding pattern and key residues involved at the interface.

**Figure 1:**
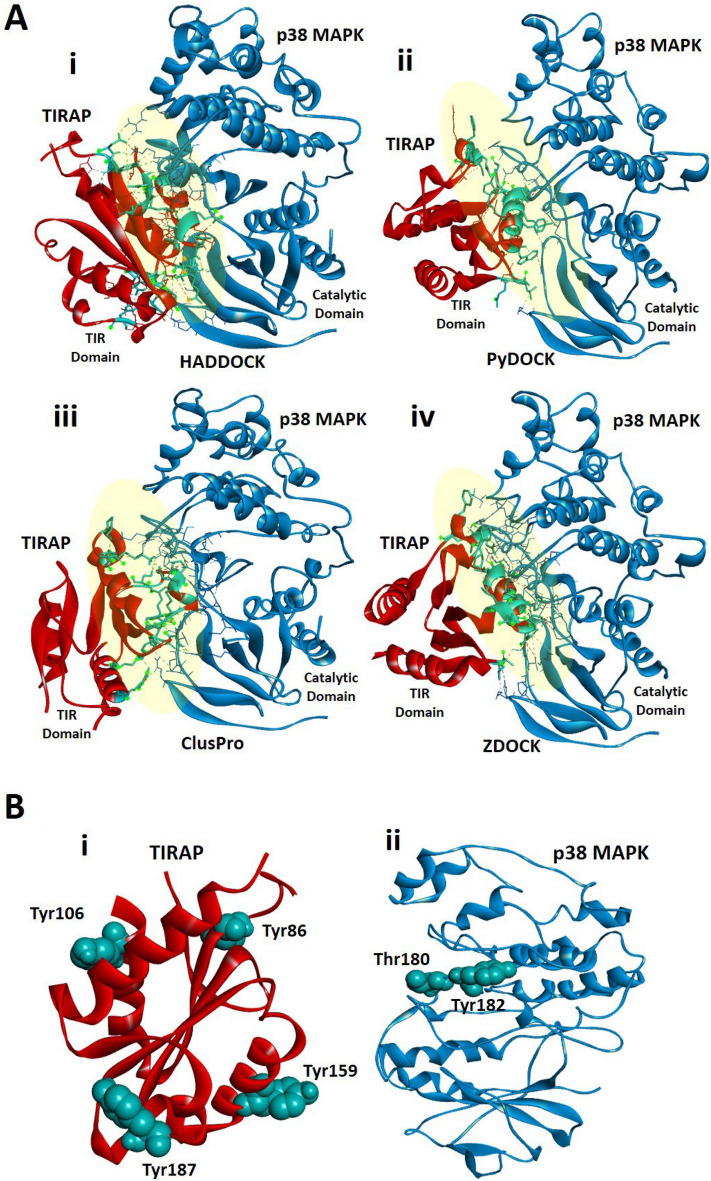
The protein–protein interaction of TIRAP and p38 MAPK. (**A**) The molecular docking complexes of TIRAP TIR domain (red) and full-length p38α MAPK protein (blue) as obtained from (i) HADDOCK 2.4 (ii) pyDockWEB (iii) ClusPro and (iv) ZDOCK 3.0.2. (**B**) Illustration of crucial tyrosine residues of (i) TIRAP TIR domain (red) Y86, Y106, Y159, and Y187, and (ii) p38 MAPK (blue) active site Y182 residue in space-filling cpk (Corey-Pauling-Koltun), respectively. The images were prepared using a Discovery studio Visualizer v21.1.0.20298.

We extensively performed the docking study of the TIRAP TIR domain crystal structure (PDB Id 3UB2) and full-length crystal structure of p38 MAPK protein (PDB Id 1WBV) (Fig. 1A). Both proteins were prepared by removing any water molecules and ligands followed by polar hydrogens addition before the protein–protein docking. First, the protein–protein docking tool HADDOCK 2.4^44^ was used for their blind docking and the topmost binding pose was analyzed. The HADDOCK 2.4 results were produced in a cluster form in order of high to low based on their Z-score with each cluster containing the four best poses. We analyzed the topmost pose from the highest-ranked cluster which was of interest since TIRAP specifically fit into the active region of p38 MAPK (Fig. 1A). Additionally, to validate the yielded binding pose of TIRAP and p38 MAPK, we included other docking methods to confirm the obtained pattern of TIRAP and p38 MAPK complex. Therefore, three more docking suits pyDockWEB^45^, ZDOCK 3.0.2^47^, and ClusPro 2.0^46^ were used for the study (Fig. 1A).

**Figure 2:**
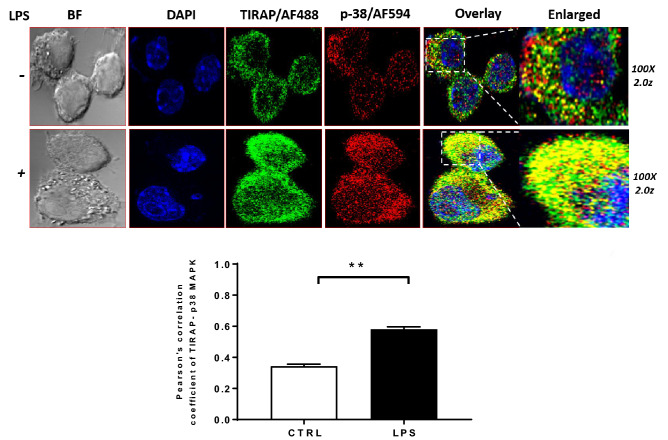
The immunofluorescence staining of TIRAP and p38 MAPK for their cellular co-localization in RAW 264.7 murine macrophages through confocal microscopy. The RAW 264.7 cells were treated with 250 ng/ml of lipopolysaccharide (LPS) for 1 h and immune-stained with mouse raised anti-TIRAP and rabbit raised anti-p38 MAPK antibody and probed with secondary antibody anti-mouse conjugated with Alexa Fluor 488 and anti-rabbit conjugated with Alexa Fluor 594. The images are captured in a confocal laser scanning microscope at 100X 2z magnification. The significant cellular colocalization of TIRAP and p38 MAPK is observed in cell cytoplasm in LPS treated cells in overlay image as compared to control cells and co-localization was quantified in ImageJ through the JACoP plugin. The data were plotted in GraphPad Prism 7 and presented as mean ± SEM. Student t-test was performed for significant difference (**p < 0.05). BF- Bright field; DAPI- 4′,6-diamidino-2-phenylindole.

Similar to the findings based on HADDOCK 2.4, only the topmost complex was selected for analysis. As a consensus, all docking produced similar binding poses for TIRAP and p38 MAPK complex suggesting that they are the best fit solution (Fig. 1A). Next to the binding pattern, the interacting residues at the interface of both TIRAP and p38 MAPK were identified from each docking result as the most favoured within the 3 Å region (Table 1). Interestingly, the interface residues revealed that the crucial tyrosine residues Y86, Y106, Y159, and Y187 occur in the TIR domain of TIRAP (Fig. 1B) whereas the p38 MAPK active site tyrosine residue Y182 (Fig. 1B) in its kinase domain is in the proximity at the interface site besides actively participating in the interaction. Further, to confirm the interaction of TIRAP and p38 MAPK at the cellular level, we performed the cellular colocalization of TIRAP and p38 MAPK in macrophages through stimulation of TLR4 signaling with LPS. The murine macrophage cell line RAW 264.7 cells were treated with 250 ng/ml of LPS for 1 h and stained with antibodies. The confocal images at 100X magnification were analyzed and interestingly we observed the significant colocalization of TIRAP and p38 MAPK in macrophages cytoplasm in LPS treated cells when compared to untreated cells (Fig. 2 and supplementary Fig. S1). Combinedly, this provided us the direct evidence of the signaling of TIRAP with p38 MAPK in the TLR4 pathway and provided us the strong evidence to understand its structural binding conformation in phosphorylated states of TIRAP. As also mentioned earlier that the previous studies found the tyrosine residues in the TIR domain to be the most important residues of TIRAP for its activation and they mainly undergo a post-translational modification of phosphorylation to impart its downstream function^17,24^. Similarly, the Y182 in the kinase domain of p38 MAPK protein, unburied and exposed outside making a surface turn, is the active site for its phosphorylation and activation making p38 MAPK catalytically active for several downstream signalling as well as transcription factor activation. In agreement with these previous studies, our docking findings for TIRAP and p38 MAPK complex undoubtedly suggested that the interaction of TIRAP in the kinase domain of p38 MAPK and the best fit model in proximity with its active residue Y182 favor it to regulate its activation and downstream function. Therefore, to establish the fact further, the effect of phosphorylation on TIRAP tyrosine residues in relation to its binding affinity and stability with p38 MAPK was analyzed.

**Table 1:** The interacting residues of TIRAP and p38 MAPK were identified using the UCSF Chimera v1.13.1 and PDBePISA within the 3 Å region.

Docking suits	TIRAP and p38 MAPK complex	TIRAP interacting and interface residues position	No. of interface residue
HADDOCK 2.4	TIRAP	D85, Y86, E94, E95, D96, L97, A100, Q101, E108, G109, S131, E132, L133, Q135, L179, S180, G181, Y187, D198, G199, R200, D203 and Y106	22
	p38 MAPK	E12, N14, K15, N26, S28, P29, S32, R49, M109, G110, D112, N114, N115, K118, C119, Q120, K152, S153, A184, R220, Y182, V183, and T185	23
pyDockWEB	TIRAP	Q135, A136, L137, W156, Y159, Q160, M161, L162, Q163, L165, T166, E167, A168, P169, G170, P189, E190, F193, and M194	19
	p38 MAPK	K15, G31, S32, G33, L171, A172, T175, D177, E178, and Y182	19
ClusPro 2.0	TIRAP	P155, K158, Y159, P169, G170, S183, R184, P189, E190, R192, M194, Y195, Y196, Q208, R215	15
	p38 MAPK	N14, K15, P29, V30, S32, G33, R49, N114, K118, D168, F169, D177, Y182, V183, and T185	15
ZDOCK 3.0.2	TIRAP	A136, L137, L152, Q153, P155, W156, K158, Y159, Q163, L165, T166, E167, P188, P189, E190, F193, and M194	17
	p38 MAPK	K15, V30, G31, S32, M109, G110, A111, D112, N114, N115, K118, F169, G170, L171, A172, D177, E178, Y182, V183, and W187	20

The p38 MAPK phosphorylation site Y182 in the kinase domain is involved in the interaction at the interface while the tyrosine-phosphorylation sites of the TIRAP TIR domain are involved on the other side.

### The cumulative effect of multi-site tyrosine phosphorylation in the TIR domain of TIRAP significantly enhances its binding affinity with p38 MAPK

We performed the site-specific *in-silico* phosphorylation of tyrosine residues of TIRAP protein to investigate its impact on the binding affinity and the conformational changes with p38 MAPK. The tyrosine residues mainly within the TIR domain of TIRAP, are imperative for its activation and downstream inflammatory signalling. The human TIR domain sequence (position 84 to 213, UniProtKB- P58753) which contains a total of six tyrosine sites (Y86, Y106, Y159, Y187, Y195, and Y196) was analyzed as confirmed by previous studies^17,25,27^, which suggests that only the first four tyrosine residues are the key sites actively involved in the phosphorylation modification to impart its function.

The four tyrosine sites (Y86, Y106, Y159, and Y187) of TIRAP are mainly investigated for their phosphorylation by the upstream tec-family kinase Bruton’s Tyrosine Kinase (BTK)as well as by the protein kinase C delta (PKCδ). Since the phosphorylation modification of the final two tyrosine residues (Y195 and Y196) is not defined experimentally and has no effect on the downstream signalling and transcription factors activation, we proposed individual phosphorylation of the four-tyrosine residues followed by the cumulative phosphorylation of all four sites to perform the docking studies with p38 MAPK. The tyrosine sites (Y86, Y106, Y159, and Y187) were phosphorylated in BIOVIA Discovery Studio 2020, adding a phosphate group to the tyrosine residues. Subsequently, the structure was energetically minimized to prepare it for docking.

**Figure 3:**
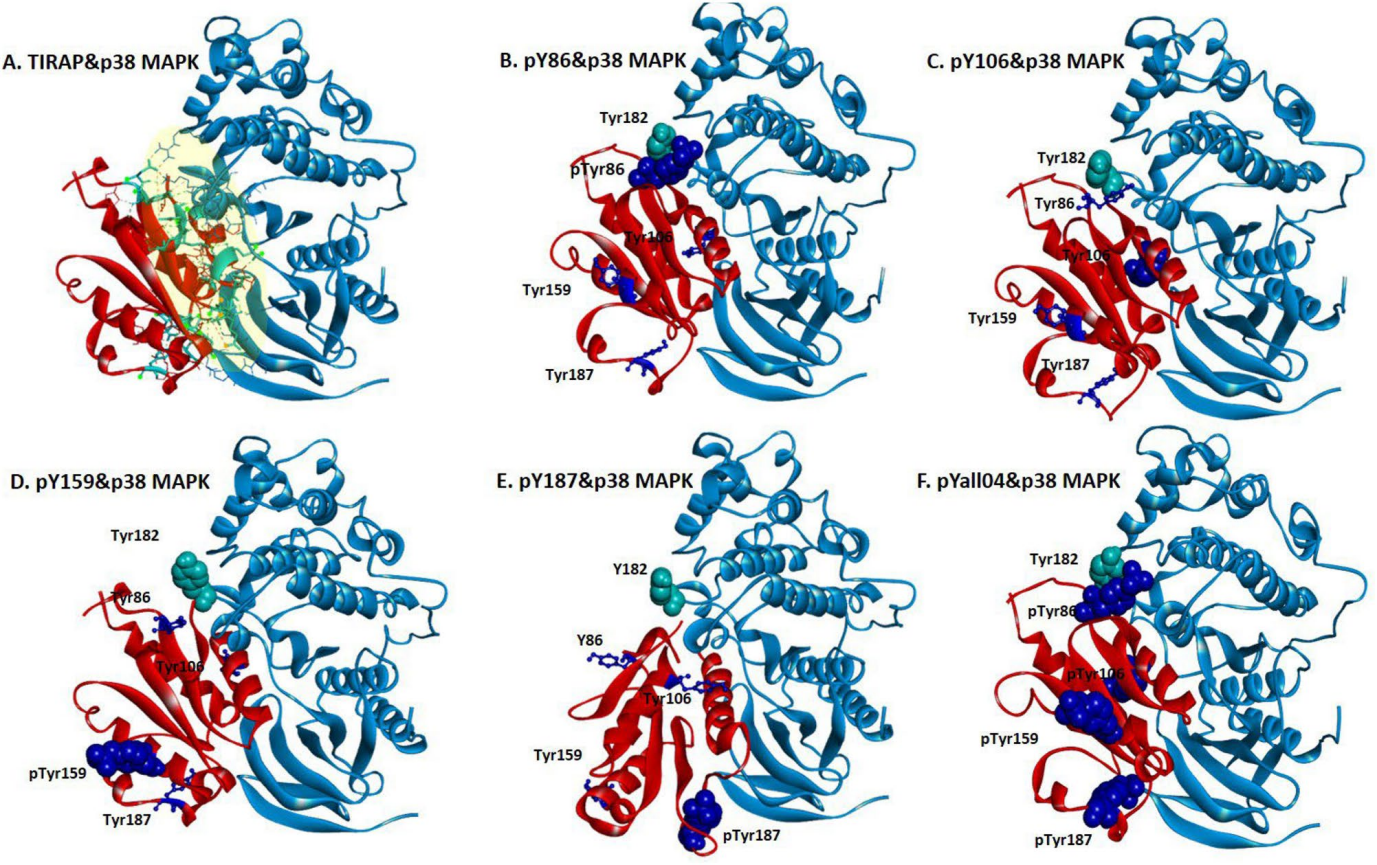
The molecular docking complex of p38 MAPK and tyrosine-phosphorylated (pY) TIRAP from the HADDOCK 2.4 tool. The complex of non-phosphorylated TIRAP (red) and p38 MAPK (light blue) (**A**) was further investigated in a comparative analysis with a docking complex of p38 MAPK (light blue) and TIRAP (red) phosphorylated tyrosine pY86 (dark blue) (**B**), pY106 (dark blue) (**C**), pY159 (dark blue) (**D**), pY187 (dark blue) (**E**) and all four phosphorylated site pYall04 (dark blue) (**F**), respectively. The complexes were the top pose result of HADDOCK 2.4 docking, and the images were prepared using a Discovery studio Visualizer v21.1.0.20298. The tyrosine residues of TIRAP (dark blue) were represented in ball and stick format whereas the phosphorylated tyrosine residue in TIRAP (blue) and p38 MAPK (cyan) were represented in cpk (Corey-Pauling-Koltun) format. The interacting interface residues between both proteins were analyzed with the 3 Å region in a Chimera v1.13.1 tool (Supplementary Table S1).

Imitating the native form, we performed the docking of tyrosine-phosphorylated TIRAP, considering it as an active protein, with non-phosphorylated p38 MAPK in the HADDOCK 2.4 tool. Previous studies have shown that phosphorylation energy can either stabilize or destabilize the structure and the differences in the binding energy by 1–2 kcal/mol is significant enough to contribute to the stabilizing or destabilizing effect on complex conformation^61,62^. Remarkably, our docking results were promising in terms of enhanced binding energy. In order to understand the effect of the energy for tyrosine phosphorylation of TIRAP with p38 MAPK interaction as compared to the non-phosphorylated TIRAP, the total binding energy (∆G_bind_) of the non-phosphorylated TIRAP and p38 MAPK complex as well as the binding energy (∆G_bind_) of phosphorylated TIRAP and p38 MAPK complex was calculated using a PDBePISA tool (Fig. 3A–F). As compared to − 10 kcal/mol (∆G_bind_) for the non-phosphorylated TIRAP and p38 MAPK complex, phosphorylation of Y86 (pY86) displayed the most significant decrease in binding energy (− 15.0 kcal/mol), followed by pY187 (− 12.0 kcal/mol), pY159 (− 11.7 kcal/mol) and pY106 (− 10.9 kcal/mol), respectively. It is evident that the phosphorylation of all these four tyrosine residues in the TIR domain of TIRAP have led to more strong binding between the complex where pY86 exerted the highest energetic effect while pY106 showed a modest effect. Multisite phosphorylation is explained to expand the regulation patterns and modulate the conformational changes more accurately whereas cooperatively increases the binding affinity to other proteins^18,63,64^. Henceforth, to confirm the effect, the cumulative response of all four phosphorylated tyrosine residues in the complex was further investigated. As expected, pYall04 TIRAP and p38 MAPK complex (Fig. 3F) produced binding energy (∆G_bind_) of − 20.5 kcal/mol suggesting the cooperative response of phosphorylated sites which attains a better conformation in the complex.

### Phosphorylated tyrosine in the TIR domain at the binding interface of TIRAP and p38 MAPK complex mainly modulates the binding conformation

The TIR domains are rather conserved in the TIR domain-containing proteins^65–67^. Similar to the TIR domain of TIRAP, most phospho-tyrosine residues are located in conserved protein domains^61,68^. However, unlike most serine and threonine sites found in the flexible region and are exposed to the surfaces of the structure, the hydrophobic tyrosine is more likely to be embedded in a structured region and phospho-tyrosine signalling are important in protein–protein interaction module^20,61^. We sought to understand the conformational dynamics of this phosphorylated tyrosine in TIRAP and p38 MAPK complexes. Interestingly, we observed that Y86 and Y187 in the TIR domain are mainly at the flexible loop of the structure when compared to two other sites (Fig. 1B). As represented in Fig. 3, in these protein–protein complex, phosphorylated Y86 conformation specifically obtains an orientation closest to the active site residues T180 and Y182 of p38 MAPK, respectively.

In contrast, the binding orientation of pY187 TIRAP with p38 MAPK complexes (Fig. 3E) was distinct from the other three complexes (Fig. 3B–D), suggesting that phosphorylation of Y187 not only brings the c-terminal loop closer to the other regions of p38 MAPK but pulls the n-terminal loop carrying Y86 distant from the active site of p38 MAPK. Moreover, the change in the free binding energy (of − 2 kcal/mol) is also marginal in phosphorylated Y187 conformation when compared to the significant decrease (− 5 kcal/mol) in the pY86 conformation from the total binding energy of non-phosphorylated TIRAP and p38 MAPK binding conformations (∆G_bind_ = − 10 kcal/mol) (Table 2A and Fig. 5). Besides, phosphorylation of Y106 and Y159 has shown no dominant effect in regulating the binding conformation of two proteins and were mainly like the pY86 complex conformation but have a marginal energetic effect which promotes a better binding of TIRAP and p38 MAPK in the phosphorylated conformation. The finding indicates the significance of Y86 phosphorylation which modulates towards a favorable complex conformation between these two protein–protein interactions and attained the highest decrease in the binding energy of the complex while all three other phosphorylated sites (Y106, Y159, and Y187) show a very close decrease in binding energy change. Nevertheless, it is notable that such effect is reversed when all four tyrosine sites of TIRAP were phosphorylated in the complex where the total binding energy (∆G_bind_ = − 20.5 kcal/mol) was comparably very much high than that of other complexes (Figs. 3F, 5, and Table 2). The interacting interface residues play imperative roles in influencing the change of such binding energy. Also, the study reports that mapping of experimentally identified phosphorylation sites on the crystal structure of human homo – and hetero- protein complexes indicated that protein interface sites are more enriched with phosphorylation sites as compared to non-interface surfaces. Additionally, such sites at the binding interfaces of hetero-protein complex exert larger changes in the binding affinity than that for other sites on the interface^18,61^. Therefore, we also analyzed the interacting residues of each complex within the 3 Å region (Supplementary Table S1) and as discussed above we found that both pY86 TIRAP and pY187 TIRAP complexes with p38 MAPK have changes in their interface residues in which the pY86 TIRAP and p38 MAPK complexes have more of the N-terminal residues including Y86 which is opposite of the pY187 TIRAP and p38 MAPK complexes in which more C-terminal residues participate in the interaction. Importantly, the interaction involved mainly pY86 and pY106 from TIRAP where T180 and Y182 from p38 MAPK were the only phosphorylation sites present among the interface residues in pY86 TIRAP and p38 complexes and pY106 TIRAP and p38 MAPK complexes, respectively (Supplementary Table S1). In the other two complexes (pY159 TIRAP and p38 MAPK and pY187 TIRAP and p38 MAPK), neither pY159 nor pY187 of TIRAP and neither T180 nor Y182 of p38 MAPK was present at the interfaces (Supplementary Table S1). Interestingly, pY86 was observed again at the interface of pYall04 TIRAP and p38 MAPK complexes (Supplementary Table S1).

**Table 2:** The energetic effect of phosphorylation and dephosphorylation of tyrosine residues (Y86, Y106, Y159, and Y187) in the TIR domain of TIRAP were calculated in terms of change in the free binding energy (BE) of TIRAP and p38 MAPK complexes.

Sr. No	TIRAP-p38 MAPK complex	Phosphorylated site(s) in TIRAP	Total BE (∆G_bind_) (kcal/mol)	Change in ∆G_bind_ (kcal/mol)
A				
1	TIRAP-p38 MAPK	–	− 10.0	–
2	p-Y86 and p38 MAPK	Y86	− 15.0	− 5
3	p-Y106 and p38 MAPK	Y106	− 10.9	− 0.9
4	p-Y159 and p38 MAPK	Y159	− 11.7	− 1.7
5	p-Y187 and p38 MAPK	Y187	− 12.0	− 2
6	p-all04 and p38 MAPK	Y86, Y106, Y159 and Y187	− 20.5	− 10.5

Sr. No	TIRAP-p38 MAPK complex	Dephosphorylated site in TIRAP	Total BE (∆G_bind_) (kcal/mol)	Change in ∆G_bind_ (kcal/mol)
B				
6	pYall04 and p38 MAPK	–	− 20.5	− 10.5
7	dpY86/pYall03 and p38 MAPK	Y86	− 7.1	− 13.4
8	dpY106/pYall03 and p38 MAPK	Y106	− 16.2	− 4.3
9	dpY159/pYall03 and p38 MAPK	Y159	− 9.6	− 10.9
10	dpY187/pYall03 and p38 MAPK	Y187	− 15.5	− 5

(A) Phosphorylation of tyrosine residues significantly decreases the binding energy (∆G_bind_) of TIRAP and p38 MAPK complex, with the highest decrease seen with pY86 while the modest seen with pY106 (B) the sequential dephosphorylation shows the highest destabilizing effect occurs with dephosphorylation of Y86 and Y159 while the modest with Y106.

The hydrogen bonds between the interfaced residues are one of the major factors contributing to the change in the binding affinity. Also at the interfaces, the phosphorylated sites contribute to the complex stability by making more hydrogen bonds than other non-phosphorylated residues on the interface. Henceforth, we further analyzed all hydrogen bonds along with their bond distances between the phosphorylated TIRAP and p38 MAPK interface residues using the PDBe PISA tool (Supplementary Table S2). In agreement with the above discussion, it was established that among all phosphorylated tyrosine, pY86 is the only one involved in the hydrogen bonding with R220 and T221 in p38 MAPK. Also, none of the three tyrosine residues present either in phosphorylated or non-phosphorylated forms formed any hydrogen bonds with p38 MAPK residues (Supplementary Table S2).

In pY86 TIRAP and p38 MAPK complexes, pY86 was found to make a single hydrogen bond with T221 within 2.89 Å. Remarkably, in pYall04 TIRAP and p38 MAPK, pY86 makes three hydrogen bonds (one very strong bond with R220 within 1.64 Å and the other two were with T221 within 3.39 Å and 3.76 Å, respectively) (Supplementary Table S2). The phenomenon strongly suggests that the presence of pY86 at the interface site largely influences the binding affinity and favors a stable conformation between TIRAP and p38 MAPK. Therefore, it is concluded that the highest increase in ∆G_bind_ in TIRAP and p38 MAPK complex is a cumulative response of all four tyrosine phosphorylation sites, although it is imperative to determine if pY86 plays any dominant roles. Hence, we further sought to investigate the destabilizing effect of these four tyrosine residues in the complex which will uncover the site-specific significance of these tyrosine residues.

### Tyrosine 86 phosphorylation (pY86) is crucial for binding affinities of TIRAP and p38 MAPK and its dephosphorylation has a destabilizing impact on the binding conformation and affinity

**Figure 4:**
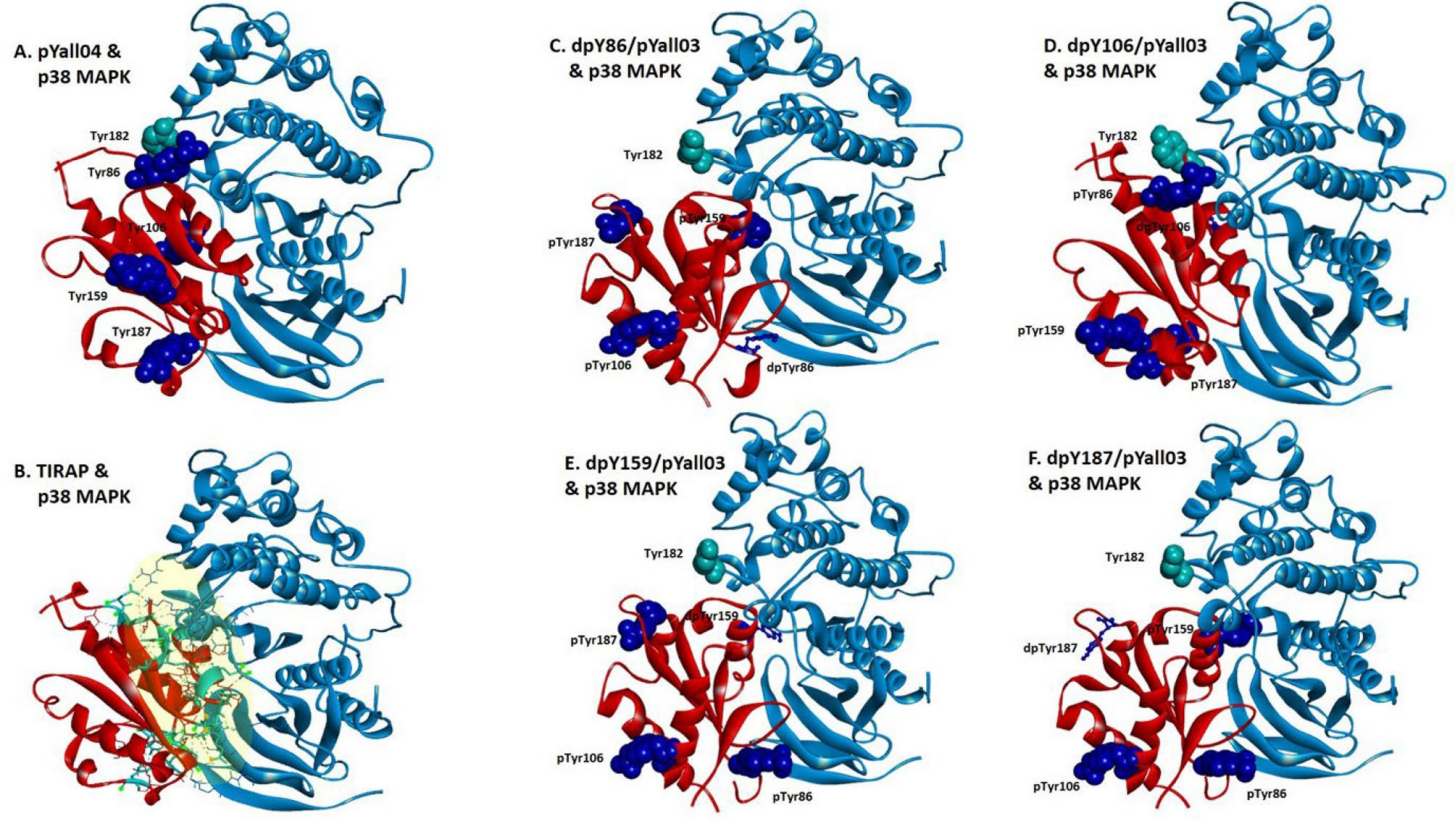
The molecular docking complex of p38 MAPK and conditionally phosphorylated tyrosine (pY) of TIRAP TIR domain from HADDOCK 2.4. The complex of non-phosphorylated and all four-tyrosine site phosphorylated TIRAP structure (red) and p38 MAPK (light blue) (**A**) and (**B**) are represented for a comparative purpose with a docking complex of p38 MAPK (light blue) and tyrosine-phosphorylated TIRAP (red) with de-phosphorylated (dp) tyrosine dpY86pYall03 (dark blue) (**C**), dpY106pYall03 (dark blue) (**D**), dpY159pYall03 (dark blue) (**E**) and dpY187pYall03 (dark blue) (**F**), respectively. The complexes are the top pose result of HADDOCK 2.4 docking and images are prepared in Discovery studio Visualizer. The tyrosine residues of TIRAP (dark blue) and p38 MAPK (cyan) are represented in a ball and stick and cpk (Corey-Pauling-Koltun) format. The images were prepared using a Discovery studio Visualizer v21.1.0.20298. The interacting interface residues between both proteins were analyzed with the 3 Å region in the Chimera v1.13.1 tool (Supplementary Table S1).

**Figure 5:**
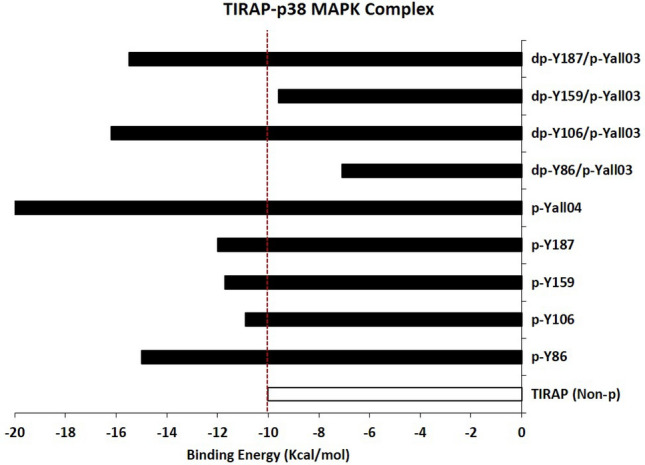
Graphical representation of the energetic effect of phosphorylation/dephosphorylation of tyrosine residues (Y86, Y106, Y159, and Y187) in TIR domain of TIRAP calculated in terms of the change in the free binding energy (BE) of TIRAP and p38 MAPK complexes. The changes in BE of phosphorylated tyrosine TIRAP and p38 MAPK complexes are compared with that for the non-phosphorylated TIRAP and p38 MAPK complex while the significance of each tyrosine residue by sequential dephosphorylation is compared with the complex of all four phosphorylated tyrosine TIRAP and p38 MAPK complexes.

In this section, we discuss the reciprocal effect of phosphorylated tyrosine sites of TIR domain in the complex of TIRAP and p38 MAPK through sequential dephosphorylation events. Earlier, we observed the conformational and energetic significance of pY86 as compared to other tyrosine sites. However, to confirm the site-specific significance of phosphorylation among the multiple study sites, we perform the sequential dephosphorylation of each tyrosine sites to calculate the destabilizing effect in terms of the adverse change in the binding energy as compared to all four phosphorylated tyrosine (pYall04) TIRAP and p38 MAPK complexes. Therefore, we prepared four different TIRAP structures each with three tyrosine sites phosphorylated (p) while one was dephosphorylated (dp) at a time [dpY86/pYall03, dpY106/pYall03, dpY159/pYall03, and dpY187/pYall03] for docking with p38 MAPK. As mentioned in the above sections, the docking was performed in the HADDOCK 2.4 tool and the yielded results were analyzed using PDBePISA and Chimera v1.13.1 tools. Again, the top pose complex of each docking results (dpY86/pYall03 and p38 MAPK, dpY106/pYall03 and p38 MAPK, dpY159/pYall03 and p38 MAPK, and dpY187/pYall03 and p38 MAPK) (Fig. 4C–F) were investigated and the total binding energy (∆G_bind_) of the complex (Table 2B and Fig. 5) calculated. As expected, pY86 has unfavorable dephosphorylation, both in terms of stabilizing effect and binding conformation. The binding conformation of TIRAP in dpY86/pYall03 and p38 MAPK was in total inverse to that for pYall04 TIRAP and non-phosphorylated TIRAP complex with p38 MAPK, respectively (Fig. 4A–C). Additionally, the total binding energy adversely changed to − 7.1 kcal/mol with a shift of − 13.4 kcal/mol when compared to the total binding energy of − 20.5 kcal/mol of pYall04 TIRAP and p38 MAPK (Table 2B). It was interesting to observe that next to dpY86, dpY159 was second to show the destabilizing effect as evident both from conformational and binding energy change (Fig. 4E and Table 2B). Meanwhile, a modest effect was observed with dephosphorylation of pY106 and pY187. In addition, there was almost no conformation change in dpY106/pYall03 and p38 MAPK (Fig. 4D). As discussed, and observed earlier about the significance of the interface residues, the change may suggest that there exists an interface whose intermolecular bonding stabilizes dpY106/pYall03 complex key residues. In contrast, despite the presence of a conformational change of TIRAP orientation in dpY187/pYall03 and p38 MAPK complexes, there was a modest destabilizing effect in the form of change in binding energy as compared to dpY86/pYall03 and dpY159/pYall03. Dephosphorylation of both pY106 and pY187 complexes of dpY106/pYall03 and dpY187/pYall03 with p38 MAPK displayed total binding energies of − 16.2 and − 15.5 kcal/mol, respectively with a slight shift of − 4.3 and − 5 kcal/mol from the total binding energy of pYall04 TIRAP and p38 MAPK complex, when compared to dpY86/pYall03 and dpY159/pYall03 (Table 2B). Therefore, this fact clearly defines the crucial role of Y86 site-specific phosphorylation in TIRAP and p38 MAPK complexes, indicating that dephosphorylation change adversely has a destabilizing effect on the complex. Secondly, Y159 phosphorylation may also be deemed critical in this complex.

Additionally, we also sought to confirm the presence of phosphorylated tyrosine at the interface residues interaction. Hence, as before, the hydrogen bonding of interface residues from each complex was analyzed. It was interesting to observe that Y86 was absent at the interface residues. Importantly, there were no hydrogen bonds found with Y86 in dpY86/pYall03 TIRAP and p38 MAPK complexes which is not what was observed with phosphorylated pY86 earlier (Table 2A and Supplementary Table S2) and it directly indicates the sharpest increase in binding energy and destabilizing effect in the complex. Moreover, the same pattern was also observed for Y159. In dpY159/pYall03 TIRAP and p38 MAPK complex too, none of the phosphorylated tyrosine nor hydrogen bonds were present at the interface. On the other hand, it was the interface phosphorylated tyrosine residues pY86 in dpY106/pYall03 and pY159 in dpY187/pYall03 complex with p38 MAPK which were present and making the hydrogen bonds. This fact not only suggests the significance of the presence of phosphorylated tyrosine at the interface but also the dominant effect of pY86 in modulating the favorable binding conformation and providing the larger binding affinity in terms of enhanced binding energy. Nevertheless, although the presence and bonding of pY159 at the interface in dpY187/pYall03 complex maintain the stabilizing effect, it fails to favor the binding conformation of TIRAP in the complex. Therefore, it is plausible that the tyrosine phosphorylation of TIRAP is crucial in the protein–protein binding to confer a better cumulative effect on the stability of the complex. However, it is pY86 that plays the most vital part in TIRAP and p38 MAPK complexes and is highly crucial for the binding conformation and affinity.

### Molecular dynamic (MD) simulation of tyrosine-phosphorylated TIRAP and p38 MAPK

**Figure 6:**
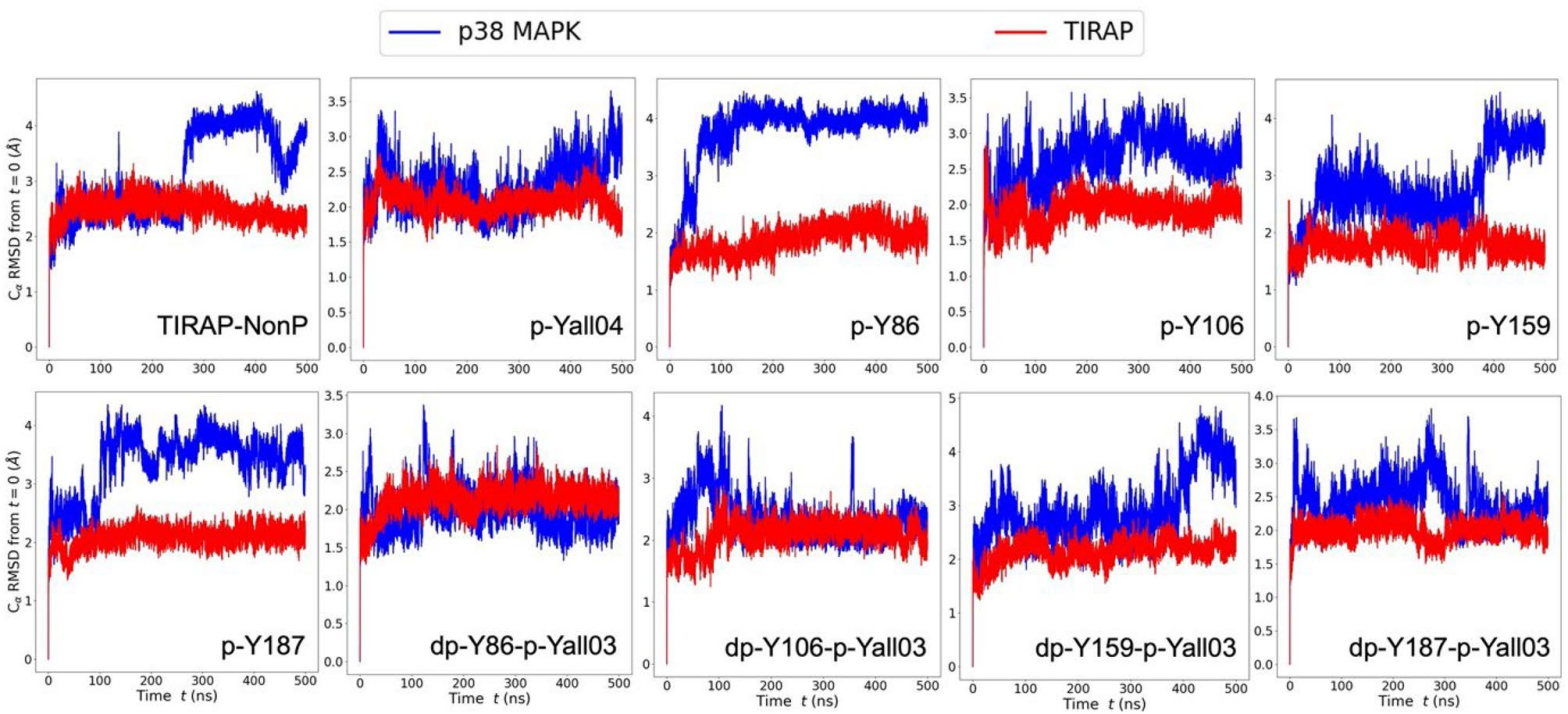
The root mean squared deviations (RMSD) of p38 MAPK and phosphorylated and non-phosphorylated TIRAP complexes. The RMSD plot of TIRAP (red) and p38 MAPK (blue) Cα atoms and their nine different molecular complexes for a total simulation time of 500 ns is shown.

**Figure 7:**
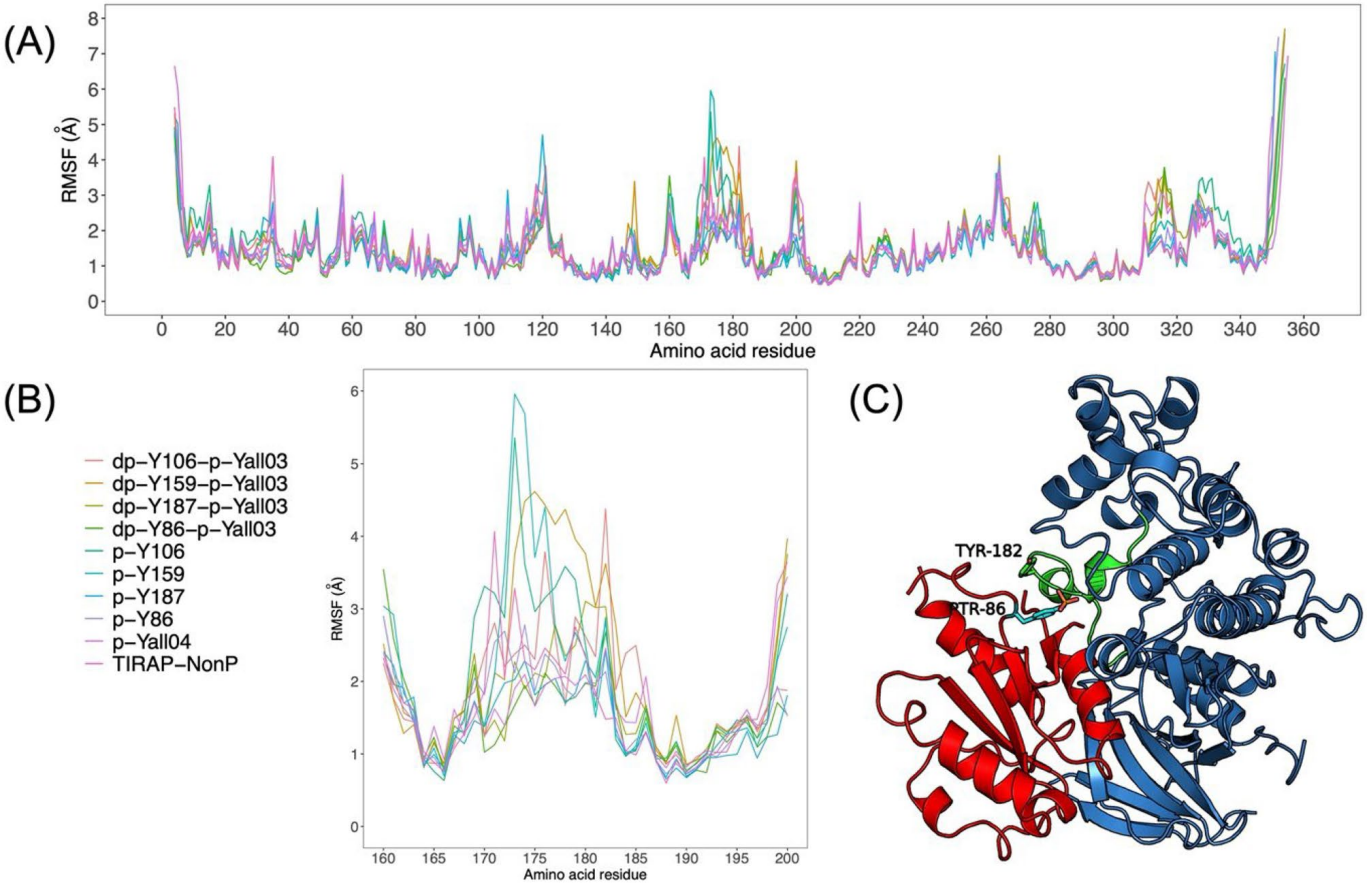
The root mean squared fluctuations (RMSF) of p38 MAPK in phosphorylated and non-phosphorylated TIRAP and p38 MAPK complexes. (**A**) RMSF of full p38 MAPK residues throughout the 500 ns MD trajectory. (**B**) RMSF of TIRAP interacting p38 MAPK residues throughout the 500 ns MD trajectory and (**C**) A cartoon diagram of TIRAP (red) and p38 MAPK (blue) complex prepared in PyMOL v2.3.4 tool showing the TIRAP interacting region (green) around the catalytic residue.

To study the effect of tyrosine phosphorylation and dephosphorylation, the docked complexes of phosphorylated and non-phosphorylated TIRAP with p38 MAPK were further analyzed using a 500 ns MD simulation employing the GROMACS program. No significant changes from the docked conformation of TIRAP were observed as suggested by small root mean squared deviations (RMSD) (Fig. 6). Comparatively higher deviations were observed for p38 MAPK mostly due to the movement of the C and N-terminal residues during dynamics (Fig. 6). Interestingly, amino acid residues at the TIRAP-p38 MAPK protein–protein interface mainly around the active site residue Y182 also account for these higher structural deviations (Fig. 7A). Specifically, in the case of pY86 TIRAP and p38 MAPK complex, conformational changes of the residues around Y182 were comparatively higher in contrast to dpY86pYall03 TIRAP and p38 MAPK complex (Fig. 7B). As these residues also form the protein–protein interface in pY86 TIRAP and p38 MAPK complex (Fig. 7C), Y86 phosphorylation in TIRAP leads to the formation of additional protein–protein contacts and thereby provided stability to the protein–protein complex.

**Figure 8:**
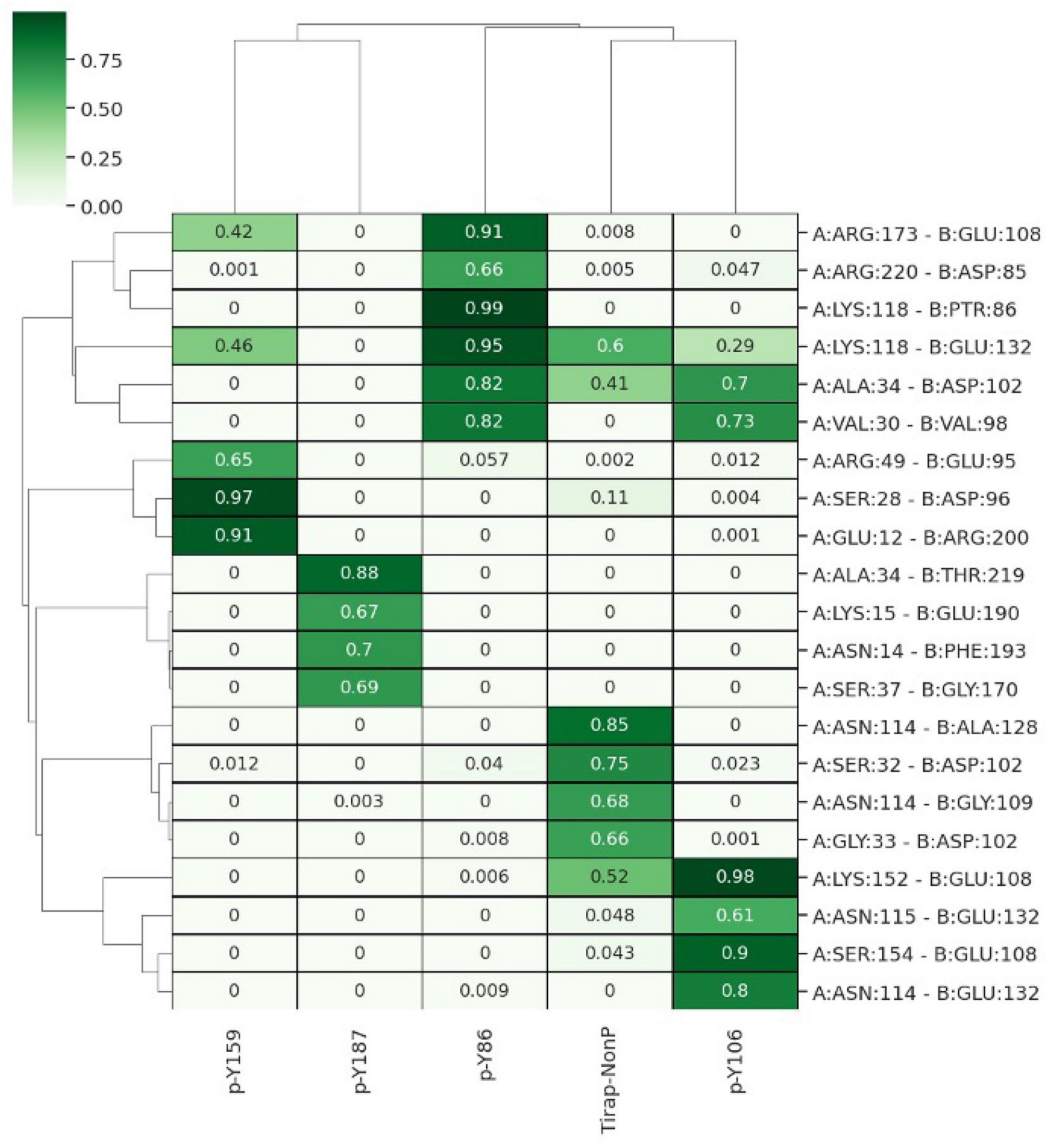
A heatmap showing the frequency of hydrogen bonding contacts between TIRAP and p38 MAPK calculated throughout the 500 ns MD trajectory. Contacts with more than 60% occurrence frequency (frequency value = 0.6) for either of the complexes are shown.

To further study the effect of tyrosine phosphorylation on TIRAP structure, PCA using Cartesian coordinates and singular value decomposition approach was performed. Analysis of the first two principal components (Supplementary Fig. S2) revealed no major conformational change. Instead, in agreement with RMSD and RMSF analysis, tyrosine phosphorylation on TIRAP structure was associated with conformational changes in p38 MAPK (Supplementary Fig. S3). As structural deviations were detected mainly in the region forming the protein–protein interface in p38 MAPK, the conformational changes might be attributed to additional contacts formed as a result of tyrosine phosphorylation in TIRAP. To analyse the dynamics of the protein–protein contacts, the frequency of all hydrogen bonds, van der Waals contacts, and salt bridges between TIRAP and p38 MAPK were calculated throughout the MD trajectory. As shown in Fig. 7, the higher number of stable hydrogen bonds (occurrence frequency > 0.8) between pY86 TIRAP and p38 MAPK complex was observed when compared to pY106, pY159, pY187, and non-phosphorylated TIRAP. A similar pattern was also observed in the case of salt bridges and van der Waals contacts (Supplementary Figs. S4 and S5). One key contact was the hydrogen bond between pY86 and K118 of p38 MAPK with almost 100% occupancy during MD (Fig. 8). It was interesting to see that instead of TIRAP’s pY86 as predicted by docking, D85 interacted with p38 MAPK residue R220 by forming hydrogen bonding and van der Waals contacts (Fig. 8 and Supplementary Fig. S5). In agreement with our docking studies, MD also highlighted the importance of phosphorylation of Y86 as being the only tyrosine among all that interacted with p38 MAPK.

In summary, taken together with the phosphorylated TIRAP interaction with p38 MAPK, it is plausible that the interaction provides a more stable conformation which significantly validates the fact that phosphorylation of tyrosine residues in the TIRAP TIR domain is crucial and greatly impacts the interaction with the downstream p38 MAPK.

## Conclusion

Overall, signalling events are tightly controlled in cellular settings and are highly regulated by several mechanisms which govern the normal functioning and maintenance of the body’s homeostasis^5,11,69^. The major event in signalling is the transduction of messenger signals to downstream molecules which mainly involves the communication between signalling mediators^15,70,71^. Protein–protein interaction which can either be a kinase-kinase or kinase-non-kinase protein interaction is one of the major events which play a crucial role between the start and the end of the signalling process. The dynamic regulations are achieved through post-translational modifications. Phosphorylation is one of the crucial post-translational modifications where the phospho-residues serine, threonine, and tyrosine regulate the function of the proteins. The importance of phospho-tyrosine residues is associated with protein–protein interaction in signalling events. Meanwhile, the presence of tyrosine residues at the binding interface has been shown to enhance the binding stability and energy of the protein complex^18–20,61^.

We have performed the structural analysis of TIRAP and p38 MAPK interaction through multiple molecular docking studies in HADDOCK 2.4, pyDockWEB, ClusPro 2.0, and ZDOCK 3.0.2, and the same has also been validated at the cellular level through *in-vitro* immunostaining study to colocalize both the proteins in murine macrophages RAW 264.7 cells. Previous studies have emphasized the crucial role of TIRAP tyrosine residues phosphorylation in TLR4/2 signaling^16,17,25^. Therefore, we further sought to understand the effect of TIRAP tyrosine phosphorylation on p38 MAPK interaction and created the in-silico site-specific phosphorylated structures of TIRAP through the discovery studio platform. Our *in-silico* data suggest that the phosphorylated Y86 of TIRAP is crucial in maintaining the structural stability of the complex since its dephosphorylation negatively impacts the binding affinity with p38 MAPK. Interestingly, phosphorylation of all four tyrosine sites jointly enhances TIRAP binding when compared to the non-phosphorylated TIRAP which is also mainly through the pY86. The phosphorylated Y86 shows to pull the TIRAP closer to the active site region in the kinase domain of p38 MAPK (T180 and Y182) while the docking of phosphorylated Y187 conformation keeps it away from the active region of p38 MAPK. Notably, the molecular docking and further the molecular simulation study of 500 ns also defines the highest stable hydrogen bonds in complex with the phosphorylated Y86 TIRAP. The structural evaluation provides an insight on the importance of tyrosine phosphorylation of TIRAP mainly at Y86 for p38 MAPK interaction. Additionally, it provides a platform for their therapeutic interventions by targeting these hydrogen bonds such as the strongest between Y86 in TIRAP and K118 in p38 MAPK for the regulation of downstream signaling and prolonged inflammatory responses responsible for several inflammatory-associated diseases.

## Supplementary Information

Supplementary Information.

## References

[1] Kumar, V. Macrophages: the potent immunoregulatory innate immune cells. *Macrophage Act.-Biol. Dis.* (2019).

[2] Oishi Y, Manabe I (2018). Macrophages in inflammation, repair and regeneration. Int. Immunol..

[3] Viola A, Munari F, Sánchez-Rodríguez R, Scolaro T, Castegna A (2019). The metabolic signature of macrophage responses. Front. Immunol..

[4] Abraham, C., Vogel, S. N. & Perkins, D. J. in *Mucosal Immunology* 605–622 (Elsevier, 2015).

[5] Beutler BA (2009). TLRs and innate immunity. Blood J. Am. Soc. Hematol..

[6] Chow JC, Young DW, Golenbock DT, Christ WJ, Gusovsky F (1999). Toll-like receptor-4 mediates lipopolysaccharide-induced signal transduction. J. Biol. Chem..

[7] Kawai T, Akira S (2006). TLR signaling. Cell Death Differ..

[8] Kawasaki T, Kawai T (2014). Toll-like receptor signaling pathways. Front Immunol..

[9] El-Zayat SR, Sibaii H, Mannaa FA (2019). Toll-like receptors activation, signaling, and targeting: an overview. Bull. Natl. Res. Centre.

[10] Takeda K, Akira S (2005). Toll-like receptors in innate immunity. Int. Immunol..

[11] Akira, S. TLR signaling. *Innate Immun. Immunol. Mem.* 1–16 (2006). 10.1007/3-540-32636-7_1 17048703

[12] O'Neill LA, Bowie AG (2007). The family of five: TIR-domain-containing adaptors in toll-like receptor signalling. Nat. Rev. Immunol..

[13] Horng T, Barton GM, Flavell RA, Medzhitov R (2002). The adaptor molecule TIRAP provides signalling specificity for Toll-like receptors. Nature.

[14] O'Neill LAJ, Fitzgerald KA, Bowie AG (2003). The Toll–IL-1 receptor adaptor family grows to five members. Trends Immunol..

[15] Rajpoot, S. *et al.* TIRAP in the Mechanism of Inflammation. 10.3389/fimmu.2021.697588 (2021). 10.3389/fimmu.2021.697588 PMC8297548 34305934

[16] Chattopadhyay S, Sen GC (2014). Tyrosine phosphorylation in Toll-like receptor signaling. Cytokine Growth Factor Rev..

[17] Piao W (2008). Tyrosine phosphorylation of MyD88 adapter-like (Mal) is critical for signal transduction and blocked in endotoxin tolerance. J. Biol. Chem..

[18] Nishi H, Shaytan A, Panchenko AR (2014). Physicochemical mechanisms of protein regulation by phosphorylation. Front. Genet..

[19] Ardito F, Giuliani M, Perrone D, Troiano G, Muzio LL (2017). The crucial role of protein phosphorylation in cell signaling and its use as targeted therapy. Int. J. Mol. Med..

[20] Kaneko T, Joshi R, Feller SM, Li SSC (2012). Phosphotyrosine recognition domains: the typical, the atypical and the versatile. Cell Commun. Signal..

[21] Songyang Z, Cantley LC (1995). Recognition and specificity in protein tyrosine kinase-mediated signalling. Trends Biochem. Sci..

[22] Wolf-Yadlin A, Sevecka M, MacBeath G (2009). Dissecting protein function and signaling using protein microarrays. Curr. Opin. Chem. Biol..

[23] Wavreille A-S, Garaud M, Zhang Y, Pei D (2007). Defining SH2 domain and PTP specificity by screening combinatorial peptide libraries. Methods.

[24] Medvedev AE (2007). Role of TLR4 tyrosine phosphorylation in signal transduction and endotoxin tolerance. J. Biol. Chem..

[25] Gray P (2006). MyD88 adapter-like (Mal) is phosphorylated by Bruton's tyrosine kinase during TLR2 and TLR4 signal transduction. J. Biol. Chem..

[26] Jefferies CA (2003). Bruton's tyrosine kinase is a Toll/interleukin-1 receptor domain-binding protein that participates in nuclear factor kappaB activation by Toll-like receptor 4. J. Biol. Chem..

[27] Jefferies CA, O'Neill LA (2004). Bruton's tyrosine kinase (Btk)-the critical tyrosine kinase in LPS signalling?. Immunol. Lett..

[28] Paracha RZ (2014). Structural evaluation of BTK and PKCdelta mediated phosphorylation of MAL at positions Tyr86 and Tyr106. Comput. Biol. Chem..

[29] Verstak B (2009). MyD88 adapter-like (Mal)/TIRAP interaction with TRAF6 is critical for TLR2- and TLR4-mediated NF-kappaB proinflammatory responses. J. Biol. Chem..

[30] Bernard NJ, O'Neill LA (2013). Mal, more than a bridge to MyD88. IUBMB Life.

[31] Fulgione A (2020). Interaction between MyD88, TIRAP and IL1RL1 against Helicobacter pylori infection. Sci. Rep..

[32] Miggin SM (2007). NF-κB activation by the Toll-IL-1 receptor domain protein MyD88 adapter-like is regulated by caspase-1. Proc. Natl. Acad. Sci..

[33] Palsson-McDermott EM, O'Neill LA (2004). Signal transduction by the lipopolysaccharide receptor, Toll-like receptor-4. Immunology.

[34] Baig MS (2017). Heterotrimeric complex of p38 MAPK, PKCdelta, and TIRAP is required for AP1 mediated inflammatory response. Int. Immunopharmacol..

[35] Bode JG, Ehlting C, Haussinger D (2012). The macrophage response towards LPS and its control through the p38(MAPK)-STAT3 axis. Cell Signal.

[36] Meng A, Zhang X, Shi Y (2014). Role of p38 MAPK and STAT3 in lipopolysaccharide-stimulated mouse alveolar macrophages. Exp. Ther. Med..

[37] Yang Y (2014). Functional roles of p38 mitogen-activated protein kinase in macrophage-mediated inflammatory responses. Mediat. Inflamm..

[38] Yong H-Y, Koh M-S, Moon A (2009). The p38 MAPK inhibitors for the treatment of inflammatory diseases and cancer. Expert Opin. Investig. Drugs.

[39] Kang YJ (2008). Macrophage deletion of p38α partially impairs lipopolysaccharide-induced cellular activation. J. Immunol..

[40] Wang Z (1997). The structure of mitogen-activated protein kinase p38 at 21-Å resolution. Proc. Natl. Acad. Sci..

[41] Kaminska B (2005). MAPK signalling pathways as molecular targets for anti-inflammatory therapy—from molecular mechanisms to therapeutic benefits. Biochim. Biophys. Acta (BBA)-Proteins Proteomics.

[42] Kumar S, Boehm J, Lee JC (2003). p38 MAP kinases: key signalling molecules as therapeutic targets for inflammatory diseases. Nat. Rev. Drug Discovery.

[43] Schindler JF, Monahan JB, Smith WG (2007). p38 pathway kinases as anti-inflammatory drug targets. J. Dent. Res..

[44] van Zundert GC, Bonvin AM (2014). Modeling protein-protein complexes using the HADDOCK webserver "modeling protein complexes with HADDOCK". Methods Mol. Biol..

[45] Jimenez-Garcia B, Pons C, Fernandez-Recio J (2013). pyDockWEB: a web server for rigid-body protein-protein docking using electrostatics and desolvation scoring. Bioinformatics.

[46] Kozakov D (2017). The ClusPro web server for protein–protein docking. Nat. Protoc..

[47] Pierce BG (2014). ZDOCK server: interactive docking prediction of protein–protein complexes and symmetric multimers. Bioinformatics.

[48] Pettersen EF (2004). UCSF Chimera–a visualization system for exploratory research and analysis. J. Comput. Chem..

[49] Biovia, D. S. Discovery Studio. *San Diego: Dassault Systemes* (2020).

[50] Schneider CA, Rasband WS, Eliceiri KW (2012). NIH Image to ImageJ: 25 years of image analysis. Nat. Methods.

[51] Bolte S, Cordelières FP (2006). A guided tour into subcellular colocalization analysis in light microscopy. J. Microsc..

[52] Foloppe N, Mackerell AD (2000). All-atom empirical force field for nucleic acids I Parameter optimization based on small molecule and condensed phase macromolecular target data. J. Comput. Chem..

[53] Abraham MJ (2015). GROMACS: high performance molecular simulations through multi-level parallelism from laptops to supercomputers. SoftwareX.

[54] Michaud-Agrawal N, Denning EJ, Woolf TB, Beckstein OJJOCC (2011). MDAnalysis: a toolkit for the analysis of molecular dynamics simulations. J. Comput. Chem..

[55] McGibbon RT (2015). MDTraj: a modern open library for the analysis of molecular dynamics trajectories. Biophys. J..

[56] Pedregosa F (2011). Scikit-learn: machine learning in Python. J. Mach. Learn. Res..

[57] Ross C (2018). MODE-TASK: large-scale protein motion tools. Bioinformatics.

[58] Hunter JD (2007). Matplotlib: A 2D graphics environment. Comput. Sci. Eng..

[59] Team, R. C. (R Foundation for Statistical Computing, Vienna, Austria, 2013).

[60] DeLano WL (2002). Pymol: An open-source molecular graphics tool. Protein Crystallogr..

[61] Nishi H, Hashimoto K, Panchenko AR (2011). Phosphorylation in protein-protein binding: effect on stability and function. Structure.

[62] Devanand T, Venkatraman P, Vemparala S (2018). Phosphorylation promotes binding affinity of Rap-Raf complex by allosteric modulation of switch loop dynamics. Sci. Rep..

[63] Kumar P (2012). Multisite phosphorylation disrupts arginine-glutamate salt bridge networks required for binding of cytoplasmic linker-associated protein 2 (CLASP2) to end-binding protein 1 (EB1). J. Biol. Chem..

[64] Ferreon JC (2009). Cooperative regulation of p53 by modulation of ternary complex formation with CBP/p300 and HDM2. Proc. Natl. Acad. Sci..

[65] Lin Z, Lu J, Zhou W, Shen Y (2012). Structural insights into TIR domain specificity of the bridging adaptor Mal in TLR4 signaling. PLoS ONE.

[66] Williams SJ (2016). Structure and function of the TIR domain from the grape NLR protein RPV1. Front. Plant Sci..

[67] Toshchakov VY, Neuwald AF (2020). A survey of TIR domain sequence and structure divergence. Immunogenetics.

[68] Sugiyama N (2008). Large-scale phosphorylation mapping reveals the extent of tyrosine phosphorylation in Arabidopsis. Mol. Syst. Biol..

[69] Kawai T, Akira S (2010). The role of pattern-recognition receptors in innate immunity: update on Toll-like receptors. Nat. Immunol..

[70] Balka KR, De Nardo D (2019). Understanding early TLR signaling through the Myddosome. J. Leukoc. Biol..

[71] Mandraju, R., Troutman, T. D. & Pasare, C. in *Reference Module in Biomedical Sciences* (Elsevier, 2014).

