# Supplementary material for: TIRAP-mediated activation of p38 MAPK in inflammatory signaling
Source: Sci Rep. 2022 Apr 4;12:5601. doi: 10.1038/s41598-022-09528-8 (PMC8979995; doi:10.1038/s41598-022-09528-8)
Supplement: Supplementary file 1 — Supplementary Information. [file 41598_2022_9528_MOESM1_ESM.docx]

**Supplementary Data**

**TIRAP-mediated activation of p38 MAPK in inflammatory signalling**

**Immunofluorescence staining of TIRAP and p38 MAPK in RAW264.7 cells*
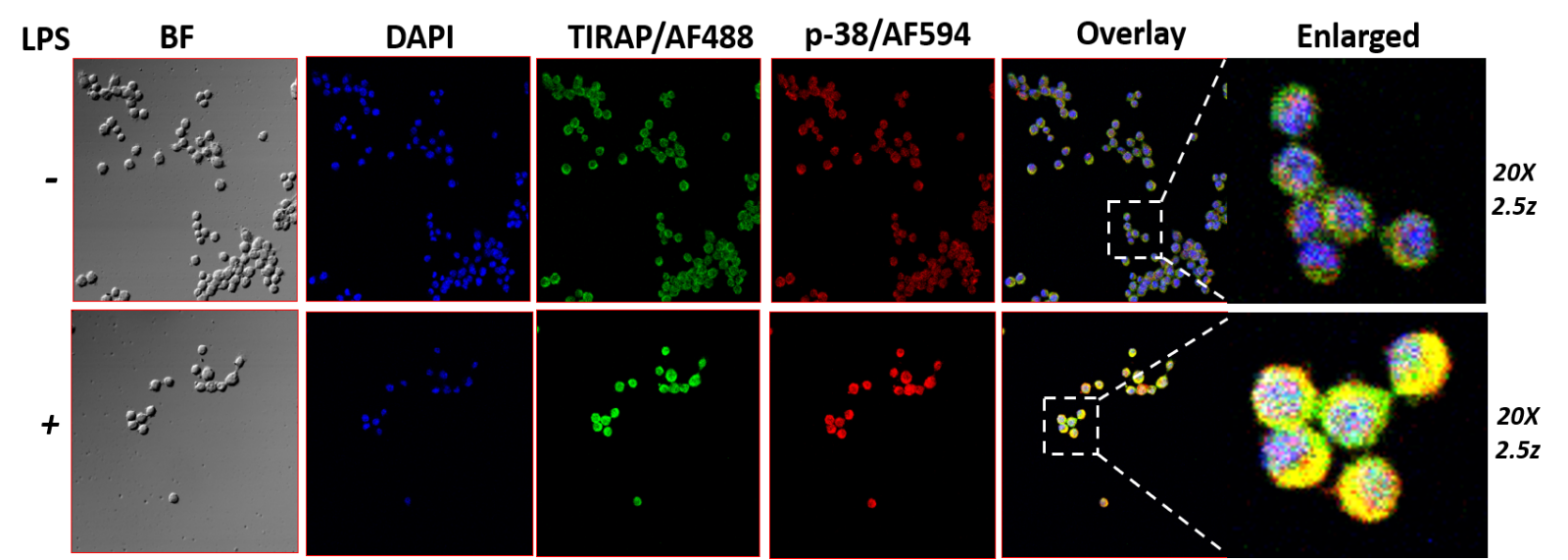
***

***Figure S1:*** ***Representation of immunofluorescence staining of TIRAP and p38 MAPK for their cellular co-localization in RAW264.7 murine macrophages through confocal microscopy.*** *The RAW264.7 cells were treated with 250ng/ml of lipopolysaccharide (LPS) for 1h and immune-stained with mouse raised anti-TIRAP and rabbit raised anti-p38 MAPK antibody and probed with secondary antibody anti-mouse conjugated with Alexa Fluor 488 and anti-rabbit conjugated with Alexa Fluor 594. The images shown are captured in confocal laser scanning microscope at 20X 2.5z magnification. The cellular colocalization of TIRAP and p38 MAPK in number of cells is observed in cytoplasm in LPS treated cells in overlay image as compared to control cells. BF- Bright field; DAPI- 4′,6-diamidino-2-phenylindole.*

**Interacting residues of TIRAP and p38 MAPK complexes**

| **Sr No.** | **TIRAP & p38 MAPK Complex** | **BE (kcal/mol)** | **Interacting and Interface Residues Position**  **Within 3Å** | | **Number of interface Residues** |
| --- | --- | --- | --- | --- | --- |
| **1.** | TIRAP-p38 MAPK | -10 | TIRAP | D85, Y86, E94, E95, D96, L97, A100, Q101, E108, G109, S131, E132, L133, Q135, L179, S180, G181, Y187, D198, G199, R200, and D203 | 22 |
|  |  |  | p38 | E12, N14, K15, N26, S28, P29, S32, R49, M109, G110, D112, N114, N115, K118, C119, Q120, K152, S153, A184, and R220 | 20 |
| **2.** | p-Y86 & p38 MAPK | -15 | TIRAP | D85, pY86, E95, V98, Q101, Y106, E108, G109, A128, I129, E132, Q135, L137, S138, P149, G181, L182, Y187, D198, G199, R200, G201, D203, G204, and F206 | 25 |
|  |  |  | p38 | N14, T16, S28, S32, G33, A34, G36, R49, S56, M109, G110, D112, K118, C119, K152, S154, T180, Y182, V183, T185, R220, and T221 | 21 |
| **3.** | p-Y106 & p38 MAPK | -10.9 | TIRAP | D85, Y86, E94, E95, Q101, V104, S105, pY106, E108, G109, A128, I129, S131, E132, Q135, S138, P149, S180, G181, L182, Y187, D198, G199, R200, D203, G204, F206, R207 | 28 |
|  |  |  | p38 | N14, S28, P29, G31, S32, G33, A34, G36, S37, R49, S56, M109, A111, D112, N114, K118, C119, K152, S154, N155, L156, A172, E178, T180, Y182, V183, A184, T185, and R220 | 29 |
| **4.** | p-Y159 & p38 MAPK | -11.7 | TIRAP | D85, Y86, EE94, E95, L97, A100, Q101, V104, S105, E108, G109, E132, L133, Q135, L137, S138, P149, G150, L179, S180, G181, D198, G199, R200, D203, and F206 | 26 |
|  |  |  | p38 | N14, K15, N26, S28, P29, S32, A34, R49, G110, N115, K118, C119, Q120, K121, K152, S154, F169, A184, and R220 | 19 |
| **5.** | p-Y187 &p38 MAPK | -12 | TIRAP | R81, S105, Y106, G109, A128, G170, A171, E172, R184, R192, F193, Y195, Y196, V197, D198, G199, R200, G201, G205, R207, Q208, K210, E211, R215, Q218, T219, and S221 | 29 |
|  |  |  | p38 | Y9, Q11, E12, N14, Q25, N26, S28, P29, S32, A34, A40, R49, A51, L108, M109, G110, N114, N115, C119, K152, S154, N155, E160, D177, and A184 | 25 |
| **6.** | p-all04 & p38 MAPK | -20.5 | TIRAP | pY86, H92, E94, E95, A99, Q101, D102, S105, E108, G109, A128, I129, E132, L133, C134, Q135, A136, P149, L179, S180, G181, pY187, G199, R200, D203, G204, G205, F206 | 28 |
|  |  |  | p38 | N14, S28, P29, V30, G31, S32, G33, A34, R49, M109, G110, N114, N115, K118, C119, K152, F169, L171, D177, Y182, A184, T185, R220, and T221 | 24 |
| **7.** | dpY86, pYall03&p38 MAPK | -7.1 | TIRAP | K84, D85, S93, E94, E95, Q135, L137, S138, S139, S140, R143, P149, G150, D154, P155, W156, C157, pY159, Q160, Q163, T166, E167, A168, E172, and G173 | 25 |
|  |  |  | p38 | Q11, L13, N14, K15, T16, N26, L27, S28, P29, V30, S32, G33, A34, S37, R49, G110, A111, N114, N115, K118, C119, Q120, and L171 | 21 |
| **8.** | dpY106, pYall03&p38 MAPK | -16.2 | TIRAP | R81, D85, pY86, E94, E95, D96, V98, Q101, S105, E108, G109, A128, I129, E132, L133, Q135, L137, S138, S180, G181, L182, pY187, R200, D203, and F206 | 25 |
|  |  |  | p38 | E12, N14, K15, S28, P29, G31, S32, A34, R49, M109, G110, N114, K118, C119, K121, K152, S154, Y182, A184, W187, R220 | 21 |
| **9.** | dpY159, pYall03&p38 MAPK | -9.6 | TIRAP | S79, S83, K84, D85, S93, E94, E95, Q135, A136, L137, H141, R143, G150, Q153, P155, W156, Y159, Q160, Q163, L165, T166, E167, A171, E172, and Y216 | 25 |
|  |  |  | p38 | Q11, E12, N14, K15, N26, L27, P29, V30, S32, G33, S37, A40, R49, M109, N114, K118, C119, Q120, and L171 | 19 |
| **10.** | dpY187, pYall03&p38 MAPK | -15.5 | TIRAP | S79, S83, K84, D85, E94, R143, P149, G150, F151, Q153, D154, P155, W156, C157, pY159, Q160, Q163, A164, T166, E167, and E172 | 21 |
|  |  |  | p38 | Q11, E12, L13, N14, K15, N26, L27, P29, V30, S32, G33, S37, G110, A111, N114, N115, K118, C119, K152, N155, G170, L171, and A172 | 23 |

***Table S1: The interacting residues of non-phosphorylated and tyrosine phosphorylated (pY) TIRAP and p38 MAPK complexes from HADDOCK 2.4.*** *The top poses were selected and interacting residues and binding energy (∆G_bind,_kcal/mol) were identified using the UCSF Chimera and PDBePISA tool within 3Å region.*

| **Sr No.** | **p38 MAPK** | **Dist. [Å]** | **pY86 TIRAP** |
| --- | --- | --- | --- |
| **1.** | A:ASN 14[HD22] | 2.47 | B:ASP 198[OD1] |
| **2.** | A:SER 32[N] | 3.85 | B:ASP 102[OD1] |
| **3.** | A:GLY 36[N] | 3.30 | B:ASP 203[OD1] |
| **4.** | A:ARG 49[HH11] | 1.53 | B:GLU 95[OE1] |
| **5.** | A:ARG 49[HH22] | 1.55 | B:GLU 95[OE2] |
| **6.** | A:ASP 112[N] | 2.96 | B:GLN 101[OE1] |
| **7.** | A:LYS 118[HZ1] | 1.57 | B:PTR 86[OP1] |
| **8.** | A:LYS 118[HZ3] | 2.32 | B:GLU 132[OE1] |
| **9.** | A:LYS 118[HZ2] | 1.75 | B:GLU 132[OE2] |
| **10.** | A:CYS 119[HG] | 2.32 | B:GLN 135[OE1] |
| **11.** | A:LYS 152[HZ2] | 2.34 | B:GLU 108[OE2] |
| **12.** | A:LYS 152[HZ3] | 2.06 | B:GLY 109[O] |
| **13.** | A:ARG 220[HH12] | 1.64 | B:PTR 86[O3P] |
| **14.** | A:ARG 220[HH11] | 2.29 | B:ASP 85[OD1] |
| **15.** | A:ARG 220[HH21] | 1.50 | B:ASP 85[OD1] |
| **16.** | A:THR 221[N] | 2.89 | B:PTR 86[O2P] |
| **17.** | A:GLY 110[O] | 3.19 | B:VAL 98[N] |
| **18.** | A:GLY 110[O] | 1.82 | B:GLN 101[HE22] |
| **19.** | A:THR 180[O] | 2.85 | B:ALA 128[N] |
| **20.** | A:TYR 182[O] | 3.01 | B:VAL 130[N] |
| **21.** | A:ASN 14[OD1] | 3.55 | B:GLY 181[N] |
| **22.** | A:ASN 14[OD1] | 3.57 | B:LEU 182[N] |
| **23.** | A:ASN 14[O] | 3.22 | B:GLY 201[N] |

| **Sr No.** | **p38 MAPK** | **Dist. [Å]** | **pY106 TIRAP** |
| --- | --- | --- | --- |
| **1.** | A:ARG 220[HH11] | 1.82 | B:ASP 85[OD2] |
| **2.** | A:ARG 220[HH21] | 1.70 | B:ASP 85[OD2] |
| **3.** | A:ARG 49[HH11] | 1.59 | B:GLU 95[OE2] |
| **4.** | A:ARG 49[HH22] | 2.25 | B:GLU 95[OE2] |
| **5.** | A:ASP 112[N] | 2.87 | B:GLN 101[OE1] |
| **6.** | A:ASN 155[HD22] | 1.95 | B:SER 105[OG] |
| **7.** | A:LYS 152[HZ2] | 1.65 | B:GLU 108[OE2] |
| **8.** | A:ALA 172[N] | 3.07 | B:GLY 109[O] |
| **9.** | A:LEU 171[N] | 3.49 | B:GLY 109[O] |
| **10.** | A:ALA 184[N] | 3.31 | B:ILE 129[O] |
| **11.** | A:ASN 114[HD22] | 2.47 | B:SER 131[O] |
| **12.** | A:LYS 118[HZ3] | 1.64 | B:GLU 132[OE1] |
| **13.** | A:LYS 118[HZ2] | 2.37 | B:GLU 132[OE2] |
| **14.** | A:GLY 36[N] | 2.85 | B:ASP 203[OD1] |
| **15.** | A:SER 154[O] | 3.85 | B:SER 105[OG] |
| **16.** | A:THR 180[O] | 2.82 | B:ALA 128[N] |
| **17.** | A:TYR 182[O] | 3.58 | B:VAL 130[N] |
| **18.** | A:PRO 29[O] | 2.96 | B:GLY 181[N] |
| **19.** | A:ASN 14[OD1] | 3.38 | B:LEU 182[N] |

**Hydrogen bonding between the interacting interface residues of TIRAP and p38 MAPK complex.**

**C D**

| **Sr No.** | **p38 MAPK** | **Dist. [Å]** | **pY159 TIRAP** |
| --- | --- | --- | --- |
| **1.** | A:ASN 14[HD21] | 2.41 | B:ASP 198[OD1] |
| **2.** | A:ASN 14[HD22] | 2.15 | B:LEU 179[O] |
| **3.** | A:SER 32[N] | 3.88 | B:GLY 204[O] |
| **4.** | A:ALA 34[N] | 2.65 | B:ASP 203[OD1] |
| **5.** | A:ARG 49[HH11] | 1.89 | B:GLU 94[OE2] |
| **6.** | A:ARG 49[HH22] | 1.77 | B:GLU 94[OE2] |
| **7.** | A:ARG 49[HH21] | 2.44 | B:GLU 94[O] |
| **8.** | A:GLY 110[N] | 3.54 | B:GLN 101[OE1] |
| **9.** | A:LYS 118[HZ3] | 1.60 | B:ASP 85[OD1] |
| **10.** | A:CYS 119[HG] | 1.65 | B:GLU 132[OE2] |
| **11.** | A:GLN 120[HE22] | 1.84 | B:GLN 135[OE1] |
| **12.** | A:LYS 152[HZ2] | 1.64 | B:GLY 109[O] |
| **13.** | A:ALA 184[N] | 2.89 | B:GLU 108[O] |
| **14.** | A:ARG 220[HH11] | 1.62 | B:ASP 85[OD2] |
| **15.** | A:ARG 220[HH21] | 1.68 | B:ASP 85[OD1] |
| **16.** | A:ASN 14[OD1] | 2.70 | B:GLY 181[N] |
| **17.** | A:ASN 14[OD1] | 3.75 | B:SER 180[OG] |
| **18.** | A:ASN 115[O] | 3.17 | B:LEU 133[N] |
| **19.** | A:LYS 118[O] | 2.45 | B:TYR 86[HH] |

| **Sr No.** | **p38 MAPK** | **Dist. [Å]** | **pY187 TIRAP** |
| --- | --- | --- | --- |
| **1.** | A:CYS 119[HG] | 1.77 | B:SER 105[O] |
| **2.** | A:GLN 11[HE21] | 1.85 | B:ARG 192[O] |
| **3.** | A:ASN 14[N] | 3.44 | B:PHE 193[O] |
| **4.** | A:ASN 26[HD21] | 1.75 | B:TYR 196[OH] |
| **5.** | A:ARG 49[HH22] | 1.98 | B:ASP 198[O] |
| **6.** | A:ARG 49[HH11] | 2.28 | B:GLY 199[O] |
| **7.** | A:ARG 49[HH12] | 2.42 | B:ARG 200[O] |
| **8.** | A::ARG 49[HH21] | 1.77 | B:GLN 208[OE1] |
| **9.** | A:ARG 49[HE] | 2.20 | B:GLN 208[OE1] |
| **10.** | A:MET 109[N] | 3.00 | B:GLU 211[OE1] |
| **11.** | A:GLY 110[N] | 2.65 | B:GLU 211[OE2] |
| **12.** | A:ASN 155[HD22] | 1.99 | B:GLN 218[OE1] |
| **13.** | A:ASN 114[OD1] | 3.71 | B:ILE 129[N] |
| **14.** | A:GLN 25[OE1] | 2.13 | B:ARG 192[HH21] |
| **15.** | A:GLU 160[OE1] | 1.53 | B:ARG 207[HH22] |
| **16.** | A:GLY 110[O] | 3.87 | B:GLU 211[N] |

**E**

| **Sr No.** | **p38 MAPK** | **Dist. [Å]** | **pYall04 TIRAP** |
| --- | --- | --- | --- |
| **1.** | A:ASN 14[HD21] | 2.19 | B:GLY 199[O] |
| **2.** | A:GLY 33[N] | 3.80 | B:ASP 203[O] |
| **3.** | A:ARG 49[HH12] | 1.98 | B:GLU 95[OE2] |
| **4.** | A:ARG 49[HH22] | 1.63 | B:GLU 95[OE2] |
| **5.** | A:ALA 111[N] | 3.45 | B:GLU 94[OE2] |
| **6.** | A:GLY 170[N] | 3.66 | B:ASP 102[OD1] |
| **7.** | A:LEU 171[N] | 2.77 | B:ASP 102[OD1] |
| **8.** | A:ALA 184[N] | 2.68 | B:GLU 108[OE2] |
| **9.** | A:THR 185[N] | 2.98 | B:GLU 108[OE1] |
| **10.** | A:THR 185[OG1] | 3.16 | B:GLU 108[OE1] |
| **11.** | A:ARG 220[HH11] | 1.64 | B:PTR 86[O3P] |
| **12.** | A:THR 221[N] | 3.39 | B:PTR 86[O2P] |
| **13.** | A:THR 221[N] | 3.76 | B:PTR 86[O3P] |
| **14.** | A:MET 109[SD] | 2.29 | B:GLN 101[HE22] |
| **15.** | A:TYR 182[O] | 3.50 | B:ILE 129[N] |
| **16.** | A:ASN 114[OD1] | 2.98 | B:LEU 133[N] |
| **17.** | A:LYS 118[O] | 3.38 | A:ALA 136[N] |
| **18.** | A:CYS 119[SG] | 3.41 | B:ALA 136[N] |
| **19.** | A:ASN 14[OD1] | 3.08 | B:GLY 181[N] |

***Table S2: Analysis of hydrogen bonding with bond distance between the interacting interface residues in tyrosine phosphorylated TIRAP and p38 MAPK docking complexes*** *(A) pY86 TIRAP & p38 MAPK, (B) pY106 TIRAP & p38 MAPK, (C) pY159 TIRAP & p38 MAPK, (D) pY187 TIRAP & p38 MAPK, and (E) pYall04 TIRAP & p38 MAPK, respectively obtained from PDBePISA tool (*[*https://www.ebi.ac.uk/pdbe/*](https://www.ebi.ac.uk/pdbe/)*).*

**Principal component analysis of tyrosine phosphorylation on TIRAP**

**
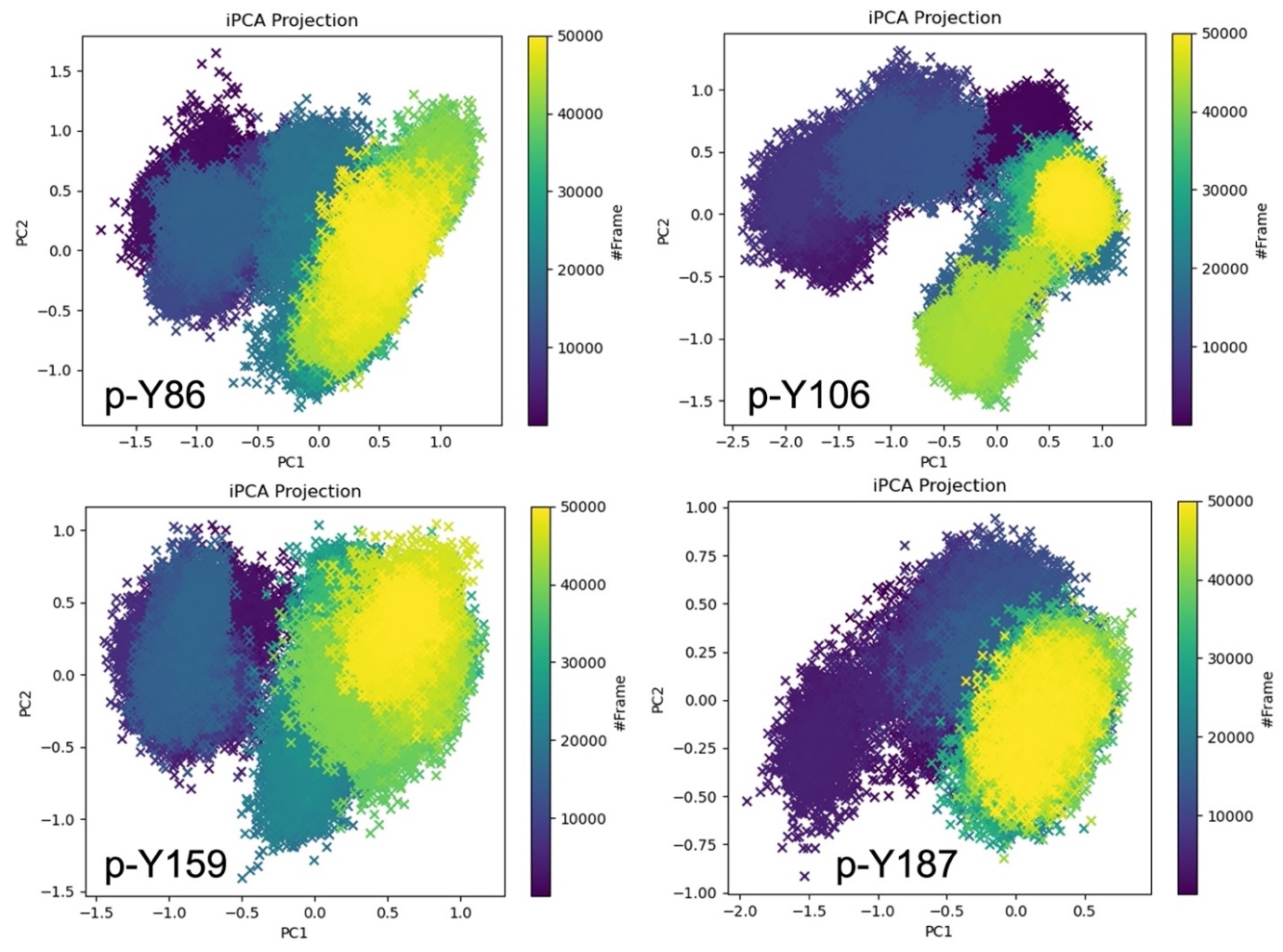
**

***Figure S2.*** *Principal component analysis to study the effect of tyrosine phosphorylation on TIRAP structure within the TIRAP p38 MAPK protein-protein complex.*

**Principal component analysis of TIRAP tyrosine phosphorylation on p38 MAPK**

***
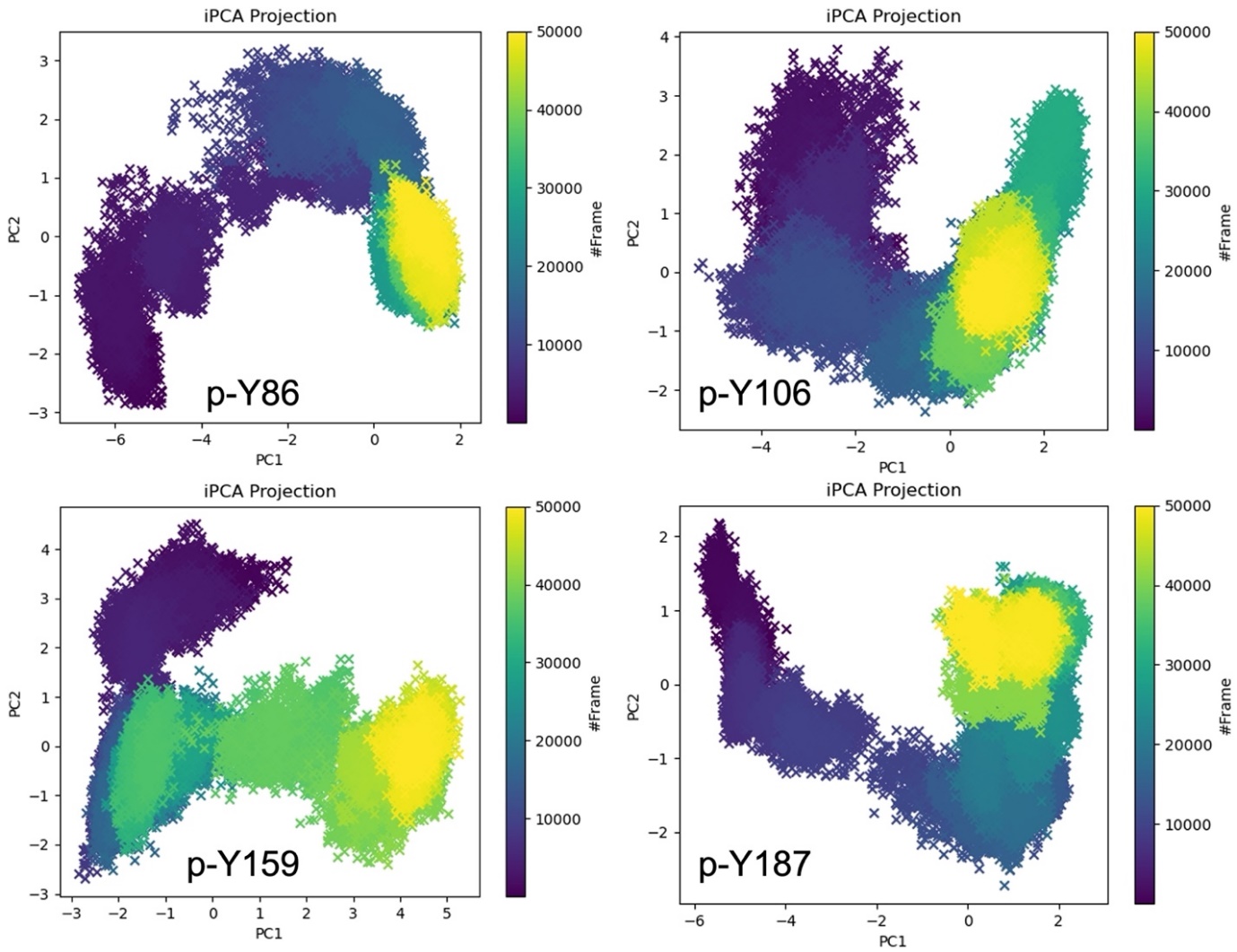
***

***Figure S3.*** *Principal component analysis to study the effect of TIRAP tyrosine phosphorylation on p38 MAPK structure within the TIRAP p38 MAPK protein-protein complex.*

**Heatmap of salt bridges between TIRAP and p38 MAPK**

***
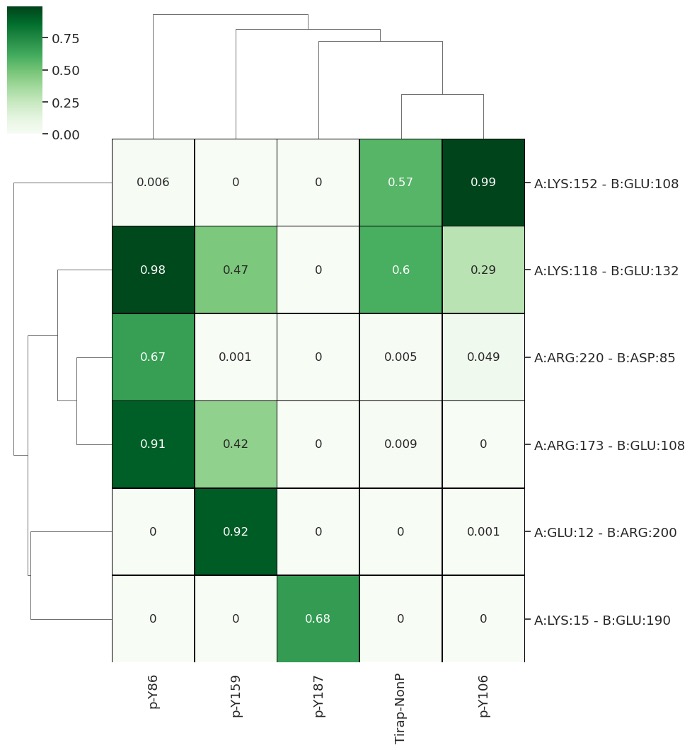
***

***Figure S4. A heatmap showing the frequency of salt bridges between TIRAP and p38 MAPK calculated throughout the 500 ns MD trajectory.*** *Contacts with more than 60% occurrence frequency (frequency value = 0.6) for either of the complexes are shown.*

**Heatmap of van der Waals contacts** **between TIRAP and p38 MAPK**

***
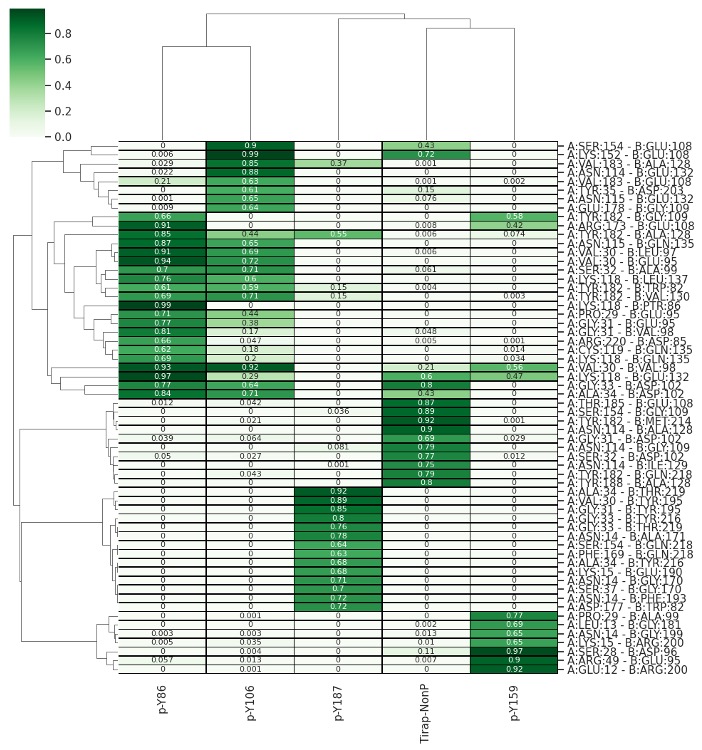
***

***Figure S5. A heatmap showing the frequency of van der Waals contacts between TIRAP and p38 MAPK calculated throughout the 500 ns MD trajectory.*** *Contacts with more than 60% occurrence frequency (frequency value = 0.6) for either of the complexes are shown.*
